# Digital Intervention Components for Preventing and Reducing Substance Use in Adolescents and Emerging Adults: A Comprehensive Scoping Review

**DOI:** 10.1007/s10935-025-00894-3

**Published:** 2026-03-20

**Authors:** Doris Ochterbeck, Saskia Muellmann, Ansgar Gerhardus, Robert Hrynyschyn, Hannah Graff, Heide Busse, Stefanie Maria Helmer

**Affiliations:** 1 Working Group Evidence-Based Public Health, Institute of Public Health and Nursing Research, University of Bremen, Bremen, Germany; 2 Department of Prevention and Evaluation, Leibniz Institute for Prevention Research and Epidemiology – BIPS, Bremen, Germany; 3 Leibniz ScienceCampus Digital Public Health, Bremen, Germany; 4 Department of Health Services Research, Institute of Public Health and Nursing Research, University of Bremen, Bremen, Germany; 5 Charité – Universitätsmedizin Berlin, Corporate Member of Freie Universität Berlin and Humboldt-Universität Zu Berlin, Institute of Health and Nursing Science, Berlin, Germany

**Keywords:** Prevention, Substance use, Adolescents, Youth, Digital, Evaluation

## Abstract

Substance use is a well-known public health problem among adolescents and emerging adults. Digital prevention components, oftentimes included in interventions to target substance use, offer unique possibilities and might be particularly suited for these age groups. However, thus far, a comprehensive overview of substance use prevention interventions utilising digital components is lacking. Hence, the overall aim of this study is to fill this research gap by identifying the type of digital components, their theoretical approaches and their combinations employed in substance use interventions for adolescents and emerging adults, and how the evidence base for their (individual) effectiveness was assessed. The literature was searched in four databases: MEDLINE, PsychInfo, CINAHL and Web of Science in May 2023. Inclusion criteria based on the PCC concept were: Population (adolescents and emerging adults aged 12–21 years), Concept ((a) digital components of interventions AND (b) prevention or reduction AND (c) substance use), Context (all contexts). Studies were selected after title/abstract screening by two independent researchers. Data extraction included: substances and substance use behaviour, underlying theory/approach, characteristics of digital intervention components, methods used for evidence assessment, and study characteristics. These data were narratively summarised. 4,905 publications were screened and 176 were included, of which 114 reported on interventions with emerging adults and 62 with adolescents. These interventions were predominantly delivered in educational settings (86%), namely universities/(vocational)schools. Alcohol was the most commonly investigated single target substance (74%). The majority of all interventions were based on more than one theory/approach and it was not always clear which theories were combined. The most commonly reported approach in interventions for adolescents was the provision of information/education, for emerging adults, interventions were based on Social Norms Theory most frequently. The majority of interventions were delivered web-based. Randomized controlled study designs dominated, and evaluation was rarely performed separately for the intervention components. A variety of digital intervention components exist to reduce or prevent substance use among adolescents and emerging adults. However, different intervention components based on diverse theoretical approaches are often combined in one intervention and the individual effects of single components remain largely unmeasured. Future studies should consider innovative methods to analyse the effectiveness of individual components, and their possible interactions, within complex interventions. Combining effective digital components or adding these components to non-digital interventions could optimize the effectiveness of interventions for young people.

**Supplementary Information:**

The online version contains supplementary material available at 10.1007/s10935-025-00894-3.

## Introduction

Substance use and addictions are a well-known public health problem (Volkow & Blanco, 2023). Alcohol, for instance, is responsible for more than 200 diseases and other adverse health conditions. It is the leading risk factor for mortality in younger age groups (Degenhardt et al., 2018; WHO, 2024).

Young people are at particular risk for substance use (Degenhardt et al., 2016). Alcohol, tobacco, and cannabis use remain prevalent among adolescents and emerging adults worldwide (WHO, 2024). Additionally, studies have indicated a notable prevalence of illicit substance use among adolescents and emerging adults (European Monitoring Centre for Drugs & Drug Addiction, 2023; Helmer et al., 2014). Substance use rates increase by age over the course of adolescence (Moor et al., 2020) and remain at a high level after leaving secondary school (Schilling et al., 2017).

Therefore, increased efforts to prevent substance use, especially among younger age groups, are required (Bryazka et al., 2022) and suitable but also innovative and scalable prevention approaches are needed (Haar et al., 2024).

As a basis for prevention, it is necessary to consider both neurobiological and psychosocial factors to understand drivers of substance use in young people. The main mechanism of action of psychoactive substances is their particular impact on the brains of users (NIDA, 2020). They cause changes in the brain chemistry, alter important brain areas such as the reward circuits, leading to compulsive substance use (NIDA, 2020). Early exposure to psychoactive substances increases the risk for developing addictions due to the greater neuroplasticity of adolescent brains (Ivanov et al., 2021; Lees et al., 2021).

In addition to these biological mechanisms, developmental and social aspects also play a decisive role. Adolescents and emerging adults find themselves in a transitional phase between adolescence and adulthood. They are engaged in forming their identity and may use substances to explore their sense of self, cope with stress or conform to peer expectations (Arnett, 2005; Steinberg, 2008). According to the Theory of Emerging Adulthood by Arnett the openness to risk behaviour during this life stage and the desire for social belonging can encourage substance use while the brain is still undergoing development (Arnett, 2005).

The rapid digitalisation of everyday life has created new opportunities for preventing and reducing substance use among young people. Digital interventions can be tailored to individual needs, adapted in pace and content, and delivered with privacy safeguards, potentially increasing engagement and effectiveness (Blanco & Volkow, 2025; O’Logbon et al., 2024). However, little is known about the theoretical models that underpin these digital interventions and if they align with the main mechanism of substance use in young people. Theories that often serve as a basis for substance use behaviour change interventions in young age groups are the Theory of Reasoned Action (Sloboda & Ringwalt, 2019), the Social Cognitive Theory, the Information–Motivation–Behavioural-Skills Model, and the Social Norms Approach (Albarracín et al., 2024). These theories often incorporate similar premises, are based on related basic assumptions and determinants (Albarracín et al., 2024). Existing reviews only offer partial insights on theoretical aspects of digital interventions, either because the focus lays on specific substances, or studies provide limited information on the theoretical basis (Johansson et al., 2024; Monarque et al., 2023; Tebb et al., 2016). Furthermore, it is not clear which theories are predominantly utilized in digital interventions on substance use.

In addition, the structural design and evaluation of digital prevention programmes are areas that remain insufficiently explored. Although many interventions employ a modular, multi-component format, they are usually evaluated as a whole. It is yet unknown which components are most effective (Collins et al., 2024).

To our knowledge, these theoretical and evaluative aspects have not been thoroughly analysed in literature reviews so far. Furthermore, most overviews focus on a single substance, primarily alcohol or nicotine (Johansson et al., 2024), specific settings such as schools or universities, or narrow age groups (Aneni et al., 2023; O’Logbon et al., 2024; Prosser et al., 2018; Strøm et al., 2014). In these reviews, comparisons of different substances, settings or age groups are thus not possible. Our review therefore has an intentionally broad scope, covering all addictive substances, both adolescents and emerging adults, and all relevant intervention settings. This is intended to provide the most comprehensive picture to date of digital approaches to substance use prevention in adolescents and emerging adults.

Specifically, the objectives of this scoping review are:

1. To provide an overview of the (digital) interventions used to prevent substance use in adolescents and emerging adults in terms of substances addressed, participant characteristics, and settings of interventions.
2. To describe the interventions in terms of digital components, the technical mode of delivery, and the theoretical background/approach of these interventions.
3. To describe which study designs and outcome variables form the methodological basis of the studies and whether the effectiveness of individual intervention components was assessed separately or in a combined approach.

## Methods

### Study Design

Scoping review methodology was employed as the work aims to identify the scope of available research, provide an overview of their focus, and map the available evidence (Grant & Booth, 2009; Munn et al., 2018, 2022). The scoping review was prospectively registered at Open Science Framework (https://osf.io/rkj98/). Conduct and reporting adhere to the methods detailed in the PRISMA (Preferred Reporting Items for Systematic Reviews and Meta Analyses) Extension for Scoping Reviews (Peters et al., 2020; Tricco et al., 2018), which is included as supplementary file S1.

### Eligibility Criteria

Since adolescents and emerging adults show high interest in digital tools (Hollis et al., 2017; Milward et al., 2016) and are also at risk for licit and illicit substance use, this scoping review focuses on these two population groups and also includes all substances and settings.

Studies were eligible for the scoping review when they met the following criteria according to the PCC (population, concept, context) scheme: (1) population: adolescents or emerging adults aged 12–21 years, (2) concept: (a) digital components of interventions AND (b) prevention or reduction AND (c) substance use, (3) context: all contexts. Furthermore, (4) original research articles reporting (5) studies of any design, (6) published in peer-reviewed journals in (7) either English or German language were included. No time-period restrictions and no restrictions on context were applied.

Exclusion criteria were defined as follows: (1) no digital components used, (2) average age of study population < 12 or > 21 years, (3) digital components not used for substance use prevention or reduction (i.e. population with diagnosed substance use disorders or in clinical/therapeutical care), (4) behavioural addictions, (5) full-text not accessible or conference abstracts without available full text, and (6) older versions of studies with updates available. Due to a high number of studies only reporting the development or subjective acceptance of interventions, two further exclusion criteria were developed that were not mentioned in the protocol (Ochterbeck et al., 2023): (7) no substance-related outcome reported (e.g. acceptance of tools only) and (8) no original research article reporting interventions (e.g. review, description of development procedure without results, study protocols).

### Study Sources

Four international bibliographic databases were searched: MEDLINE (via PubMed), PsychInfo, CINAHL, and Web of Science.

### Search Strategy

The search syntax was developed by one researcher (DO) with the support of an experienced librarian. It was designed for MEDLINE and subsequently adapted to the other databases (detailed syntax in supplementary files S2-S5). On 26th (MEDLINE, CINAHL) and 27th (PsychInfo, Web of Science) May 2023, the search was conducted (by DO). All records were exported to Zotero 6.0.36 (Roy Rosenzweig Center for History and New Media, Fairfax County, USA) for deduplication with manual control.

### Study Selection

For screening, the records were exported from Zotero to Rayyan software (Rayyan Systems Inc., Cambridge, USA) (Ouzzani et al., 2016). Titles and abstracts were independently screened by at least two researchers (DO and HB/AG/RH/SMH/SM) in a blinded process. After solving conflicts by discussion until consensus was reached, full texts were retrieved and subsequently screened (blinded) by at least two screeners (DO and AG/HG/RH/SMH/SM). In cases of conflicts, final consensus was reached either by discussion (two screeners) or by a majority decision (three screeners).

### Data Charting

One researcher (DO) developed the data extraction sheet in Microsoft Excel 2019 (Microsoft Corp., Redmond, USA) and extracted all relevant data. All entries were checked for correctness and completeness by a second researcher (AG/HG/RH/SMH/SM). Conflicts were solved in discussion until consensus was reached. Besides bibliographical information and study details (authors, year of publication, country of study conduct, sample sizes at baseline and latest follow-up, number of study arms, comparison groups), the following items were extracted from the included studies:

- *Overview of interventions:* (1) target substance, (2) consumption patterns of participants (heavy users/mandated students only, users only, or all, inclusive of abstainers), and other inclusion criteria specifications, (3) implementation setting, and (4) average age of participants at baseline.
- *Theoretical approach, delivery, and components of intervention*: (1) theory/approach underlying the intervention as mentioned in the publication, (2) (technical) mode of delivery, and (3) components of digital interventions. Interventional approaches aiming at substance use prevention are commonly based on specific theoretical constructs or incorporate active ingredients grounded in a theoretical basis. The respective item category ‘theory/approach’ in this review thus comprises specific theoretical models (e.g. Social Norms used in Personalised Normative Feedback (PNF) or Social Cognitive Theory), therapeutic models (e.g. skills training, cognitive bias modification), and intervention techniques (e.g. Motivational Interviewing (MI), harm reduction techniques) (Monarque et al., 2023; Tebb et al., 2016). Often only specific approaches such as the provision of information/education are mentioned in studies and are therefore coded in an overarching category.
- *Evidence assessment:* (1) outcome measures, (2) study design, (3) combined or separate evaluation of multi-component interventions.

To map the two age groups (adolescents and emerging adults), the data extraction sheet was split in two, and the respective analyses were conducted for each age group separately. The average age (at baseline) in the samples from 12.0 to 17.7 years was set for adolescents, and an average age of 17.8 to 21.0 years was assigned to emerging adults.

## Results

### Study Selection

A total of 7,482 records were identified in the four databases. Following the removal of duplicates (n = 2,577), 4,905 remaining publications were eligible for title abstract screening. In the course of this process, n = 4,448 further records were excluded, leaving n = 457 for full text screening. Of these, 281 were excluded with justification, leaving 176 studies reporting 185 interventions that were included in the scoping review.

**Fig. 1:**
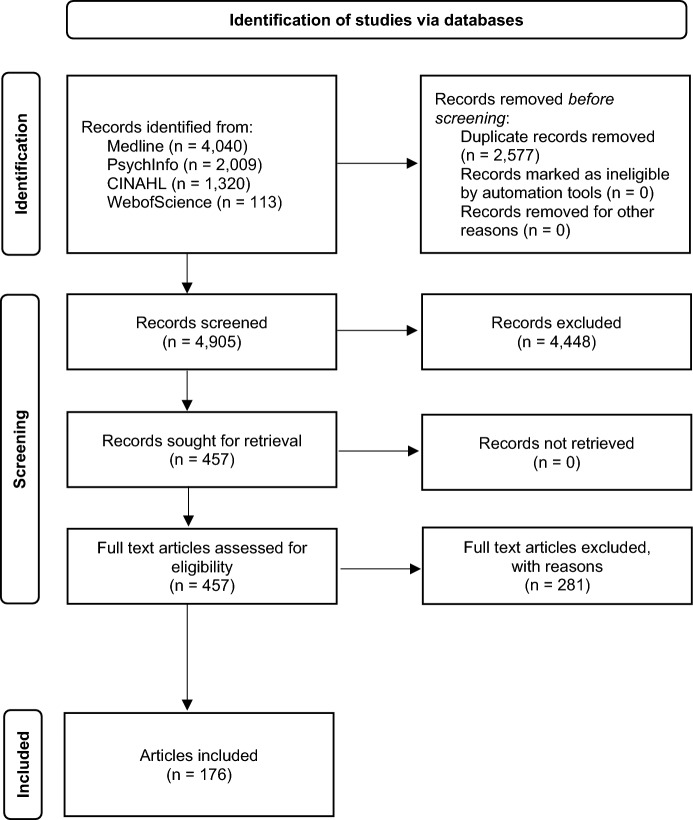
Search process: PRISMA flow chart. From Page MJ, McKenzie JE, Bossuyt PM, Boutron I, Hoffmann TC, Mulrow CD, et al. The PRISMA 2020 statement: an updated guideline for reporting systematic reviews. BMJ 2021;372:n71. 10.1136/bmj.n71. For more information, visit: http://www.prisma-statement.org/

An overview of the search process, based on the PRISMA flow chart (Tricco et al., 2018), is illustrated in Fig. 1.

### Characteristics of Included Studies

In the 176 included articles, emerging adults were addressed in 114 (65%), while 62 (35%) focussed on adolescents.

Studies from 20 different countries were identified. The majority of studies were conducted in the United States of America (n = 127, 69%) followed by Australia (n = 14, 8%) and Switzerland (n = 11, 6%). Sample sizes ranged from a minimum of 9 to a maximum of 35,104 participants at baseline (mean: 1,090, median: 317), and from 9 to 33,644 at the latest follow-up (mean: 846, median: 247). The follow-up periods comprised a range of zero months (immediately after the intervention) up to a maximum of 84 months (mean: 6, median: 3). Further details, also on study arms and comparison groups, are collated in Supplementary Files S6, S9 and S10 (Table 1).

**Table 1:** Included studies

Interventions		Publications
*A. Adolescents*		
1	Alc-Check	Haug et al. (2013)
2	Alcohol Alert/Alerta Alcohol	Jander et al. (2016), Martinez-Montilla et al. (2020), Vargas-Martinez et al. (2019)
3	C2Q-Teen (Craving to Quit-Teen)	Pbert et al. (2020)
4	Click City®: Alcohol	Gordon et al. (2017)
5	Climate Schools	Champion et al. (2016) and Newton et al. (2022b)
6	Climate Schools	Newton et al. (2022a) and Vogl et al. (2009)
7	Climate Schools Psychostimulant & Cannabis Modlue	Vogl et al. (2014)
8	Climate Schools: Ecstasy and Emerging Drugs Module	Champion et al. (2018)
9	eCHUG (electronic version of Check-up to go)	Doumas and Esp (2019), Doumas et al. (2020a, 2020b, 2021)
10	E-health4Uth	Bannink et al. (2014)
11	Elm City Story (PlayForward)	Montanaro et al. (2015)
12	E-MOVO (Dutch for Electronic Monitoring and Health Promotion)	Crutzen et al. (2008)
13	Girls and Stress	Schinke and Schwinn (2005)
14	Health4Life	Champion et al. (2023)
15	Healthy School and Drugs	Malmberg et al. (2014)
16	HSD (Healthy School and Drugs)	Koning et al. (2011)
17	Invite Only VR (virtual reality)	Weser et al. (2021)
18	kiR (keepin’ it REAL)	Shin et al. (2018)
19	MobileCoach Alcohol	Haug et al. (2017b, 2020, 2023)
20	P4T (POP4Teens) vs JTT (JustThinkTwice)	Marsch et al. (2021)
21	PALMSS (peer alcohol knowledge, low-risk drinking, media-influence, social drinking, self-efficacy)	Hongthong and Areesantichai (2016)
22	Pure Rush	Stapinski et al. (2018)
23	QuitSTART smoking app	Pbert et al. (2020)
24	¿Qu é pasa si te pasas? (What happens if you go to far?)	Fuentes et al. (2023)
25	ready4life	Haug et al. (2022)
26	REAL media	Greene et al. (2020)
27	RealTeen	Schwinn et al. (2019, 2020)
28	Refuse to Use	Duncan et al. (2000)
29	Smart Choices 4 Teens	Byrnes et al. (2019)
30	SmartCoach	Paz Castro et al. (2022)
31	smokeSCREEN	Bteddini et al. (2023)
32	SPORT	Mathews et al. (2007)
33	Vamos	Schwinn et al. (2021)
34	VR *FestLab* (virtual reality FestLab)	Guldager et al. (2022)
35	WDYD (What do you drink?)	Voogt et al. (2013)
36	WISEteens	Arnaud et al. (2016)
37	xkpts.com ((reader: “perquèpetes.com”, translation: “why joints?”)	Ariza et al. (2013)
38	Your Decision Counts TM	Evers et al. (2012)
39	*No name (family interaction)*	Schinke et al. (2009a, 2011)
40	*No name*	Fang and Schinke (2013)
41	*No name*	Gryczynski et al. (2021)
42	*No name*	Ho et al. (2021)
43	*No name*	Knight et al. (2018)
44	*No name*	Knight et al. (2019)
45	*No name*	Kong et al. (2017)
46	*No name*	Mason et al. (2015)
47	*No name*	Russell et al. (2017)
48	*No name*	Schinke et al. (2009b)
49	*No name*	Schwinn et al. (2015)
50	*No name*	Shi et al. (2013)
51	*No name*	Williams et al. (2005)
*B. Emerging adults*		
1	Alcohol 101	Barnett et al. (2007), Braitman and Henson (2016), Carey et al. (2009)
2	Alcohol 101 Plus™	Carey et al. (2011) (study 2)
3	Alcohol Edu® for College	Croom et al. (2009), Hustad et al., 2010 (A) and Paschall et al. (2014)
4	Alcohol Edu® for Sanctions	Carey et al. (2011) (study 1)
5	Alcohol-Wise	Croom et al. (2015) (study 1 & 2), Gilbertson et al. (2018) and Strohman et al. (2016)
6	alcooquizz.ch (1)	Bertholet et al. (2015, 2016, 2018)
7	BASICS (Brief Alcohol Screening and Intervention for College Students—adaption for marijuana)	Lee et al. (2010)
8	BASICS (Brief Alcohol Screening and Intervention for College Students)	Lee et al. (2014), Linowski et al. (2016), Murphy et al. (2010), (study 1&2), Patrick et al. (2014, 2021), Saitz et al. (2007)
9	BASICS (Brief Alcohol Screening and Intervention for College Students)—PNF component	LaBrie et al. (2013), Larimer et al. (2021), Moreira et al. (2012), Neighbors et al. (2004, 2006, 2010)
10	CAAT (Cannabis Approach Avoidance Training)	Jacobus et al. (2018)
11	CAAT (Cannabis Approach Avoidance Training)	Karoly et al. (2019)
12	Campus GANDR (Gamified Alcohol Norm Discovery & Readjustment)	Boyle et al. (2017)
13	CDCU (College Drinker's Check-up)	Carey et al. (2017), Hester et al. (2012) (study 1&2)
14	CIAN (Call it a Night®)	Fucito et al. (2017)
15	College Alc	Bersamin et al. (2007)
16	D_ARIANNA ((Digital-Alcohol Risk Alertness Notifying Network for Adolescents and Young Adults))	Carrà et al. (2016)
17	DrAFT-CS (Drinking Assessment and Feedback Tool for College Students)	Wagener et al. (2012) and Weaver et al. (2014)
18	d-SBI (Digital screening and brief intervention for alcohol misuse)	Sharma et al. (2023)
19	ECALC (expectancy challenge alcohol literacy curriculum)	Dunn et al. (2020) (B) 2019?
20	eCHUG (electronic version of Check-up to go)	Alfonso et al. (2013), Braitman et al. (2022), Doumas et al. (2009), Hustad et al. (2010 (B), Thompson et al. (2018), Tahaney and Palfai (2017) and Walters et al. (2007)
21	eCHUG (electronic version of Check-up to go) marijuana	Elliott et al. (2014) and Palfai et al. (2014b)
22	eentjeteveel	Fraeyman et al. (2012)
23	eSBI (electronic screening and brief intervention)	Kypri et al. (2008, 2013, 2014)
24	ETUCARE	Theurel et al. (2022)
25	FIT (functional imagery training)	Shuai et al. (2022)
26	iHeLP	Braciszewski et al. (2018)
27	Meine Zeit ohne—Die Challenge	Pietsch et al. (2023)
28	MiSARA (substance abuse research assistant)	Coughlin et al. (2021)
29	Mobile Coach Tobacco & Mobile Coach Tobacco +	Haug et al. (2017a)
30	M-PASS (Michigan Prevention and Alcohol Safety for Students)	Bingham et al. (2011)
31	myPlaybook (alcohol)	Fearnow-Kenney et al. (2016) and Zamboanga et al. (2019)
32	myPlaybook (alcohol & cannabis)	Rulison et al. (2022 (study 1, 2, 3)
33	MyStudentBody:Alcohol	Chiauzzi et al. (2005)
34	PantherTRAC	Suffoletto et al. (2016)
35	PartyWise	Schwarz et al. (2022)
36	Project Fitness	Moore et al. (2012)
37	Ray's Night Out	Hides et al. (2018) and Pocuca et al. (2016)
38	THRIVE (Tertiary Health Research Intervention via Email)	Kypri et al. (2009) and Leeman et al. (2016)
39	U@Uni:LifeGuide	Cameron et al. (2015)
40	Unitcheck	Bewick et al. (2013) and Marley et al. (2016)
41	WDYD (What do you drink?)	Voogt et al. (2014)
42	Y1-CAP (Year 1 College Alcohol Profile)	Shell and Newman (2019)
43	No name	Bernstein et al. (2018)
44	No name	Bonar et al. (2022)
45	*No name*	Boyle et al. (2021)
46	*No name*	Braitman and Lau-Barraco (2020)
47	*No name*	Bryant et al. (2013)
48	*No name*	Buckner et al. (2019)
49	*No name*	Buckner et al. (2020)
50	*No name*	Butler and Correia (2009)
51	*No name*	Carey et al. (2020)
52	*No name*	Collins et al. (2014)
53	*No name*	Doumas et al. (2011)
54	*No name*	Ellis et al. (2017)
55	*No name*	Gex et al. (2023)
56	*No name*	Glowacki et al. (2020)
57	*No name*	Hagger et al. (2012)
58	*No name*	Hendershot et al. (2010)
59	*No name*	Kazemi et al. (2020 (study 1)
60	*No name*	Kazemi et al. (2020 (study 2)
61	*No name*	LaBrie et al. (2019)
62	*No name*	Larimer et al. (2023)
63	*No name*	Leary et al. (2022)
64	*No name*	Leavens et al. (2020)
65	*No name*	Lewis et al. (2007)
66	*No name*	Ma (2022)
67	*No name*	Mason et al. (2014)
68	No name	McGeary et al. (2014)
69	*No name*	Neighbors et al. (2009)
70	*No name*	Norman et al. (2018)
71	*No name*	Palfai et al. (2011)
72	*No name*	Palfai et al. (2014a)
73	*No name*	Pilling and Brannon (2007)
74	*No name*	Ridout and Campbell (2014)
75	*No name*	Riordan et al. (2023)
76	*No name*	Savage et al. (2015)
77	*No name*	Schuckit et al. (2016)
78	*No name*	Smallman et al. (2023)
79	*No name*	Spijkerman et al. (2010)
80	*No name*	Tello et al. (2018)
81	*No name*	Thombs et al. (2007)
82	*No name*	Walters et al. (2009)
83	*No name*	Walukevich-Dienst et al. (2019)
84	*No name*	Whitt et al. (2019)
85	*No name*	Yang and Nan (2019)

#### Substances and Settings

##### Adolescents

The most common single substance targeted by digital prevention of substance use among adolescents was alcohol (n = 25 studies, 40%). The majority of these studies addressed all adolescents, irrespective of their consumption patterns, whereas 16% (n = 4) focussed on either heavy users (n = 2, 8%), or users in general (n = 2, 8%). Twenty-five studies (40%) targeted several substances simultaneously. Of these, 52% (n = 13) focussed on heavy users only, 8% (n = 2) on users in general, and 40% (n = 10) were directed at adolescents in general.

Sixty-six percent (n = 41) of the interventions were implemented in schools and 3% (n = 2) in vocational schools. The remaining 18% (n = 11) were delivered to the general population with no specific location, 8% (n = 5) in families, 3% (n = 2) in primary care settings, and 2% (n = 1) at a community organisation (supplementary file S7).

##### Emerging Adults

Alcohol was by far the most commonly addressed single substance, accounting for 106 (86%) of the studies. Of these, 51 studies (48%) included only heavy users or mandated students, 23 studies (22%) studies excluded emerging adults who never drank (users only), and 32 studies (30%) included all young people, regardless of their consumption patterns. Cannabis interventions were tested in six studies (5%), of which one included only heavy users or ‘mandated students’ (those who had violated regulations and therefore received conditions), four of which were directed at users, and another one addressed all emerging adults. One study each (1%) addressed tobacco users and heavy users of a substance of choice. Finally, nine studies (7%) investigated interventions to prevent the use of a combination of substances such as alcohol and cannabis (n = 6 heavy users or mandated students, n = 1 users, n = 2 all) (supplementary file S7).

The vast majority of interventions for emerging adults (n = 107, 87%) was carried out at universities. The remainder were implemented in vocational schools (n = 2, 2%) and on a general population level without focussing on a specific setting (n = 14, 11%) (Table 2).

**Table 2:** Settings and target substances

Setting	Target substance	Publications
A Adolescents		
School	Alcohol	Jander et al. (2016), Martinez-Montilla et al. (2020), Vargas-Martínez et al. (2019), Gordon et al. (2017), Newton et al. (2022a), Vogl et al. (2009), Doumas and Esp (2019), Doumas et al. (2020a, 2020b, 2021), Koning et al. (2011), Shin et al. (2018), Haug et al. (2017b, 2023), Mathews et al. (2007), Guldager et al. (2022), Ho et al. (2021), Hongthong and Areesantichai (2016), Haug et al. (2013, 2020), Voogt et al. (2013)
	Tobacco, incl. E-cigarettes	Weser et al. (2021), Pbert et al. (2020), and Shi et al. (2013)
	Cannabis	Ariza et al. (2013)
	Exstasy & emerging drugs	Champion et al. (2018)
	Alcohol & cannabis	Champion et al. (2016), Newton et al. (2022b), and Gryczynski et al. (2021)
	Alcohol & tobacco	Crutzen et al. (2008) and Champion et al. (2023)
	Alcohol, cannabis & tobacco	Malmberg et al. (2014), Fuentes et al. (2023), Paz Castro et al. (2022) Haug et al. (2022)
	Other drugs & combinations	Bannink et al. (2014), Evers et al. (2012), Schinke and Schwinn (2005), Williams et al. (2005), Duncan et al. (2000) and Stapinski et al. (2018) and Vogl et al. (2014)
General population	Alcohol	Russell et al. (2017)
	Tobacco, incl. E-cigarettes	Bteddini et al. (2023), Mason et al. (2015) and Kong et al. (2017)
	Other drugs & combinations	Montanaro et al. (2015), Arnaud et al. (2016), Schwinn et al. (2010, 2015, 2019, 2021)
Family	Alcohol	Byrnes et al. (2019) and Schinke et al. (2009b)
	Other drugs & combinations	Schinke et al. (2009a, 2011) and Fang and Schinke (2013)
Primary care	Alcohol	Knight et al. (2018)
	Alcohol & cannabis	Knight et al. (2019)
Community organisations	Drug use	Greene et al. (2020)
B Emerging adults		
University	Alcohol	Braitman and Henson (2016), Croom et al. (2009), Hustad et al. (2010) (A), Paschall et al. (2014), Croom et al. (2015) (study 1), Croom et al. (2015) (study 2), Gilbertson et al. (2018), Strohman et al. (2016), Patrick et al. (2014, 2021), Larimer et al. (2021), Moreira et al. (2012), Boyle et al. (2017), Bersamin et al. (2007), Hustad et al. (2010), Thompson et al. (2018), Fraeyman et al. (2012), Kypri et al. (2013), Theurel et al. (2022), Shell and Newman (2019), Bryant et al. (2013), Glowacki et al. (2020), Hagger et al. (2012), Hendershot et al. (2010), Ma (2022), Norman et al. (2018), Palfai et al. (2014a), Pilling and Brannon (2007), Riordan et al. (2023), Tello et al. (2018), Thombs et al., 2007, Carey et al. (2011), Barnett et al. (2007), Carey et al. (2009, 2011), Weaver et al. (2014), Kypri et al. (2014), Bingham et al. (2011), Fearnow-Kenney et al. (2016), Bewick et al. (2013), Bernstein et al. (2018), Braitman and Lau-Barraco (2020), Boyle et al. (2021), Buckner et al. (2019), Ellis et al. (2017), Kazemi et al. (2020) (study 2), Leary et al. (2022), Neighbors et al. (2009), Savage et al. (2015), Smallman et al. (2023), Whitt et al. (2019), Yang and Nan (2019), Lee et al. (2014), Linowski et al. (2016), Murphy et al. (2010) (study 1), Murphy et al. (2010) (study 2), Saitz et al. (2007), LaBrie et al. (2013), Neighbors et al. (2004, 2006, 2010), Carey et al. (2017), Hester et al. (2012) (study 1), Hester et al. (2012) (study 2), Fucito et al. (2017), Wagener et al. (2012), Dunn et al. (2020), Alfonso et al. (2013), Braitman et al. (2022), Doumas et al. (2011),Tahaney and Palfay (2017), Walters et al. (2007), Kypri et al. (2008, 2009), Chiauzzi et al. (2005), Suffoletto et al. (2016), Pocuca et al. (2016), Leeman et al. (2016), Marley et al. (2016), Voogt et al. (2014), Butler et al. (2009), Carey et al. (2020), Collins et al. (2014), Doumas et al. (2009), Gex et al. (2023), Kazemi et al. (2020) (study 1), LaBrie et al. (2019), Larimer et al. (2023), Leavens et al. (2020), Lewis et al. (2007), Mason et al. (2014), McGeary et al. (2014), Palfai et al. (2011), Ridout and Campbell (2014), Schuckit et al. (2016) and Walters et al. (2009)
	Tobacco	Haug et al. (2017a)
	Cannabis	Elliott et al. (2014), Lee et al. (2010), Palfai et al. (2014b), Buckner et al. (2020) and Walukevich-Dienst et al. (2019)
	Alcohol & tobacco	Cameron et al. (2015)
	Alcohol & cannabis	Rulison et al. (2022) (study 2 & study 3)
	Other drugs & combinations	Moore et al. (2012), Rulison et al. (2022) (study 1), Pietsch et al. (2023), and Sharma et al. (2023)
General population	Alcohol	Schwarz et al. (2022), Zamboanga et al. (2019), Hides et al. (2018), Bertholet et al. (2015, 2016, 2018), Carrà et al. (2016), Shuai et al. (2022), Bonar et al. (2022) and Spijkerman et al. (2010)
	Cannabis	Karoly et al. (2019)
	Alcohol & cannabis	Jacobus et al. (2018) and Coughlin et al. (2021)
	Other drugs & combinations	Braciszewski et al. (2018)

#### Theory/Approach, Delivery, and Components

##### Adolescents

For adolescents, more than 90% of all studies combined different theories/approaches for the interventions. Most commonly information/education (n = 31, 50%) and harm reduction strategies (n = 29, 47%) were included in the interventions. Social Norms Theory was underlying in 32% (n = 20) of the interventions, Furthermore, 27% (n = 17) of the studies used MI. Social Cognitive Theory was mentioned in 2% (n = 15) as underlying theory. In 19% (n = 12), other theories/approaches were included (supplementary file S8). Digital interventions were reported to be based on a single theory or approach only provided use information/education (n = 4, 6%) or Social Norms Theory (n = 2, 3%).

The technical delivery of the interventions in adolescents was mainly web-based (n = 39, 63%). Other deliveries mentioned included: apps (n = 12, 19%), CDs (n = 11, 18%), computers (n = 7, 11%), text messages (n = 6, 10%), and head-mounted devices (n = 2, 3%).

The most common component of the interventions was information/education content (n = 35, 56%). Furthermore, frequently used features were personal messages/feedback (n = 21, 34%), non-interactive videos (n = 17, 27%), interactive videos (n = 13, 21%), exercise/training (n = 12, 19%), and games (n = 11, 18%). In 21 studies (34%), the interventions were complemented by non-digital components (Supplementary file S8) (Table 3).

**Table 3:** Intervention delivery, components and underlying theory by target group (n of studies)

Intervention delivery										
	Web-based	PC/computer-based	CD	App (phone or web or both)	Facebook	Head mounted device (hmd)	Text message	Mail	Single method	Combination of methods
Adolescents	39	7	11	12	0	2	6	0	29	48
Emerging adults	88	12	0	9	2	1	12	10	107	31

Components of digital interventions															
	Information/education	Non-interactive video	Interactive video	Virtual reality 3D (hmd)	Avatar/interactive video	Chatroom/discussion with peers	Chatroom/discussion with experts	Personal message/feedback	Chatbot	Quiz	Contest	Games	Exercise/training/tasks	Other	Additional non-digital components
Adolescents	36	17	14	2	0	1	3	21	1	10	2	11	12	6	22
Emerging adults	73	1	11	1	11	2	3	85	0	9	2	5	12	1	6

Underlying theory									
	Personal normative feedback	Motivational interviewing	Info/education	Harm prevention techniques	Social cognitive theory	Transtheoretical model	Heath belief model	Theory of planned behaviour	Others
Adolescents	20	17	31	29	15	2			10
Emerging adults	98	35	37	37	2	6	5	5	13

##### Emerging Adults

In studies with emerging adults, nearly 75% (n = 91) of all studies were based on several theories/approaches. The minority of interventions (n = 32, 26%) were reported to be based only on a single theory/approach (Social Norms Theory: n = 30, information/education: n = 1, harm prevention strategies: n = 1). Overall, Social Norms Theory (used in PNF) was mentioned as one theoretical concept for about 80% (n = 98) of digital interventions. The provision of information/education and harm prevention strategies were also included in the interventions, accounting for 30% each (n = 37). MI was used in 28% (n = 35). In about 25% of the studies (n = 31) other theories/approaches were mentioned. These can be found in supplementary file S8.

The technical delivery of most interventions was reported to be web-based (n = 88, 72%). Other deliveries mentioned included: computers (n = 12, 10%), apps (n = 9, 7%), text messages (n = 12, 10%), emails (n = 10, 8%), Facebook (n = 2, 2%), and head-mounted devices (n = 1, 1%).

The components primarily integrated in the digital interventions were personal messages/feedback (n = 86, 70%) and information/education content (n = 73, 59%). Furthermore, exercise/training (n = 12, 10%), interactive videos (n = 11, 9%), avatar-based interactive videos (n = 11, 9%), and quizzes (n = 9, 7%) were part of the digital interventions. Six studies (5%) also included non-digital components such as classroom sessions (supplementary file S8).

#### Evidence Assessment

##### Adolescents

The following outcome measures were used to assess the effectiveness of interventions in adolescents: Consumption (n = 48, 77%), binge/heavy drinking (n = 25, 40%), intention to use (n = 16, 26%), knowledge (n = 15, 24%), perception of peer use (n = 14, 23%), self-efficacy measures (n = 13, 21%), norms and attitudes (n = 12, 19%), and protective behavioural strategies (n = 11, 18%). Substance-related problems (n = 8, 13%), motivation/readiness to change (n = 5, 8%), and consumption effects expectancies (n = 3, 5%) were reported comparatively less frequently. In 24 studies (39%), further outcome measures, e.g. help-seeking, family history & age of consumption onset were used.

Studies of interventions in adolescents were predominantly conducted in RCTs and Cluster RCTs (n = 53, 85%). Seven studies (11%) applied a pre-post design, and two studies (3%) reported other designs, for example a paired intervention-control design.

Three single-component interventions were reported (5%). The other interventions comprised of several components and accounted for 95% (n = 59). All interventions were evaluated as a whole, with no consideration of individual intervention components.

##### Emerging Adults

Evidence for the effectiveness of interventions in emerging adults was assessed with a range of outcome measures. Usually, multiple outcome measures were combined. The most common outcome was consumption of substances, which was assessed in 110 studies (89%), followed by binge/heavy drinking (n = 70, 57%), substance-related problems (n = 69, 56%), perceived peer use (n = 35, 28%), protective behavioural strategies (n = 25, 20%), motivation/readiness to change (n = 21, 17%), norms and attitudes (n = 19, 15%), intention to use (n = 18, 15%), consumption effects expectancies (n = 14, 11%), self-efficacy (n = 5, 4%), and knowledge (n = 2, 2%). Further, more particular outcome measures such as risk behaviour, adaption to college, other health-related behaviour, or details on family consumption history were applied in n = 48 (39%) of all studies (supplementary files S9 & S10).

Digital interventions in emerging adults were predominantly studied in RCTs and Cluster RCTs (n = 109, 89%). Four studies (3%) applied a pre-post design, two studies (2%) used a qualitative approach, and eight studies (7%) reported further designs (supplementary files S9 & S10).

The largest proportion of digital interventions which consisted of several components (n = 96, 76%) were evaluated as a whole (n = 85). In 11 multi-component-studies, the components were regarded separately, such as by factorial designs that allow the analysis of effects of individual components as well as the interactions between components.

## Discussion

This scoping review investigated the characteristics of digital interventions to prevent or reduce substance use in young people aged 12–21 years across a total of 176 included studies. Alcohol was the most common single substance targeted in the included studies. The majority of all interventions were implemented in educational settings (schools and universities), and consisted of different intervention components based on diverse theoretical approaches. The effectiveness of interventions was mostly evaluated as a whole package.

The finding that the majority of digital interventions were implemented in educational **settings** is in line with other reviews in the field (Biallas et al., 2022). Setting-based interventions are commonplace when focussing on substance use as well as for other behaviour-change interventions, such as nutrition and physical activity in younger age groups (Brandes et al., 2022; Chatterjee & Nirgude, 2024; Jago et al., 2023; Karpouzis et al., 2025). They allow easy reach of the target group and enable a combination of behaviour- as well as settingmodifying components. Other settings, such as communities, were used rarely for substance use prevention, possibly due to difficulties in implementation and sustainability in these environments (Stockings et al., 2016).

About two thirds of the included papers focussed on emerging adults, and about one third on adolescents. This could be due to the fact that fewer ethical and administrative hurdles exist to data collection. For example, parental consent must necessarily be obtained for minors to participate in studies. Furthermore, teachers in schools may be reluctant to address substance use too early and are therefore unwilling to implement appropriate measures in younger age groups. They may fear that they will achieve the opposite of the desired goal by encouraging consumption out of curiosity. Studies amng teachers’ opinions on this issue would provide clarity here. However, given the high vulnerability of the adolescent brain, as well as habit formation in early adolescence (Ivanov et al., 2021; Lees et al., 2021), a stronger focus on younger age groups needs to be discussed. Nevertheless, it is unclear whether mainly non-digital interventions are being implemented in schools, which are not part of the present review. Further studies are required to determine this.

The **substances** targeted by digital prevention of substance use differed between age groups. While the majority of trials for emerging adults targeted alcohol use alone, in adolescents either alcohol or a combination of several substances were addressed likewise in interventions. The focus on alcohol and sub-groups such as heavy users aligns with the actual use among adolescents and emerging adults, as alcohol remains the most commonly consumed substance and heavy drinking also remains prevalent (Helmer et al., 2021; Kraus et al., 2018; Tholen et al., 2025). Addressing different substances or poly-substance use within interventions for adolescents also corresponds with research that shows that the majority of adolescents’ report using two substances or more (Zuckermann et al., 2020). However, changes within the consumer scene are to be expected, for instance with regard to e-cigarette use and the non-medical use of pharmaceutical drugs (European Union Drugs Agency, 2025). It is thus important to further monitor the consumption patterns age-specifically, and develop interventions tailored to this, or adapt existing ones accordingly.

Our review additionally showed, that **theoretical underpinnings and intervention mechanisms** were not always clearly described which is in line with other research (Prestwich et al., 2015; Tebb et al., 2016). However, there is no clear consensus on the benefits of theory-based interventions, with some studies suggesting they are more effective, while others have found contradictory results (Prestwich et al., 2015). This may be due in part to the fact that theories are not always clearly and effectively implemented in interventions (Prestwich et al., 2015). We did not, however, focus on the development process of the interventions included in the review. This requires further reviews with a corresponding focus.

Furthermore, multiple theories were often combined in multi-component interventions and the sole effectiveness of components based on a specific theory could not be clearly described which is consistent with the literature (Das et al., 2016). In this case it is questionable whether specific conclusions can be drawn about the significance of single components to preventing substance use.

One specific theory that was mostly mentioned as a sole basis for interventions was the Social Norms Theory, which was also mentioned as a prominent interventional approach for young people in the literature (Haug et al., 2012). Peers are known as important reference group for adolescents and emerging adults and are recognized as one of the most important influence factor for substance use in the literature (Helmer et al., 2021; Nawi et al., 2021). However, even though the theoretical concept of the Social Norms approach suits the target group, the evidence base for this approach is not entirely clear and meta-analytic research shows only small effects on substance use reduction (Foxcroft & Tsertsvadze, 2011; Saxton et al., 2021). As another intervention approach, information/education was often used to expand the knowledge base of participants. The effectiveness of this component is also considered negligible in empirical studies even though it is intended as a principle of behavioural change (Albarracín et al., 2024).

Mostly, the **technical delivery** was indicated as web-based followed by apps and text-messages. An analysis of digital tools for health promotion in general found that social media is frequently used (Ferretti et al., 2023) which only played a minor role in our included studies. This might be explained by the fact that the majority of interventions were implemented in educational settings that may not foresee an integration of social media components.

A striking difference between the age groups concerns the integration of non-digital components, which occurred relatively frequently in programmes for adolescents, but rarely in those for emerging adults. Regarding the technical components, interactive elements, exercises, quizzes, and games were offered in the minority of programmes. Supported by findings from Ferretti et al.’s scoping review, who found that a lack of human interaction was seen as weaknesses of interventions, the integration of interactive elements, possibly in a co-creative design process, is warranted (Ferretti et al., 2023).

Overall, most of the interventions were **multi-component interventions evaluated** as a whole by RCTs or Cluster RCTs. Despite the growing use of digital interventions in the field of substance use prevention, RCTs and Cluster RCTs remain the primary research design for demonstrating causal relationships between interventions and outcomes (Freidlin & Korn, 2012; Thiese, 2014; Victora et al., 2004). However, the challenges of evaluating digital interventions through RCTs have become increasingly apparent. Longer study durations of RCTs coincide with shorter development and update cycles of digital interventions and present problems in the evaluation of digital interventions using RCTs. In addition, there are problems between the rigid study protocols of RCTs and the more flexible and context-specific characteristics of digital interventions (Moor et al., 2020). Additionally, to address challenges in the evaluation of digital interventions, alternative evaluation methods such as stepped-wedge designs, sequential multiple assignment trials (SMART) and n-of-1 designs were developed, but are still rarely employed (Hrynyschyn et al., 2022). These alternative evaluation methods could help to facilitate a more nuanced understanding of the mechanisms through which these interventions exert their effects.

Moreover, using RCTs lead to the effect that multi-component interventions were evaluated in a combined form, not accounting for the effects of single components. The treatment package approach in RCTs is widely used in behavioural interventions but has limited informative value since the approach does not allow researchers to identify which components are effective and which are redundant or even counterproductive (Collins, 2018; Collins et al., 2007).

### Strengths and Limitations

The overall strength of this study is that it provides a comprehensive overview of a comparatively large number of studies using digital components on a pressing issue of continuing relevance to public health – substance use by adolescents and emerging adults. However, scoping reviews have some drawbacks as a research methodology as they do not provide a formal quality assessment (Grant & Booth, 2009; Munn et al., 2018, 2022; ; Peters et al., 2015). Especially the in-depth evaluation of the effectiveness of the interventions would require a systematic review approach. However, we have included the respective authors’ assessment of the effectiveness of their interventions in the course of data extraction, and have reported this. Further limitations of this study include the possibility that we missed relevant studies, even though four major databases were searched. Also, studies in languages other than English and German were not included. In addition, clustering approaches and theories that served as a basis for interventions was necessary, as not all publications mentioned a theoretical model such as PNF or MI. Others provide information about the underlying therapeutical approaches and/or intervention techniques. Therefore, categories were built that included theories as well as approaches.

### Implications for Research and Practice

The majority of the interventions in this review were implemented in the educational setting, which is in line with research findings investigating other behaviour-change interventions (Brandes et al., 2022; Chatterjee & Nirgude, 2024; Jago et al., 2023; Karpouzis et al., 2025). Accessibility in particular is best ensured here. The future inclusion of interventions in school curricula could be beneficial, as well as the investigation of teachers’ feedback on implementation details (Karpouzis et al., 2025). However, other settings such as communities should be treated as a priority in the future to reach also adolescents and emerging adults that are not part of the named educational settings.

Interventions targeting emerging adults primarily focused on alcohol use, whereas those for adolescents addressed either alcohol alone or a combination of multiple substances. Given the tendency to legitimise cannabis and the rise of use of new psychoactive drugs and e-cigarettes, this focus needs to be critically evaluated and possibly expanded.

Furthermore, it should be investigated in future studies which types of interventions are best suited to young target groups. Here, it would be interesting to examine the extent to which a combination of digital and non-digital is appropriate for young people. Furthermore, cultural adaptions for traditionally culturally underserved groups were rarely included in the studies described in this review and need to be developed in future studies (Nittas et al., 2024).

For both age groups investigated here, the interventions were informed by a variety of theories and approaches. Logic models to understand hypothesised pathways, however, are widely lacking. Also, further research focusing on the design processes of interventions, based on a framework such as the Behavioural Change Wheel (Vasiliou et al., 2021), could provide additional information on how these can best be adapted to different target groups, including those with different consumption patterns, for example.

Furthermore, the majority of interventions consisted of multiple components and were evaluated collectively using (Cluster) RCTs. We found that even when interventions consisted of several components, these were rarely assessed separately. This indicates an urgent need for studies that use innovative methods to analyse the effectiveness of single components as well as their possible interactions. This could mean that conducting them in an iterative design, implementation, and optimisation process, which also includes analyses in the course of the implementation phase, could be of particular value.

## Conclusions

A range of digital intervention components aiming to reduce or prevent substance use among adolescents and emerging adults were identified in this review. These components are often based on different theories and were commonly bundled within an intervention package, making it difficult to determine the specific impact of each component. Future research should apply innovative methods to evaluate how individual components contribute to effectiveness of interventions. Integrating the most effective digital elements, or adding them to non-digital programs, may enhance outcomes for young people.

## Supplementary Information

Below is the link to the electronic supplementary material.

Supplementary file1 (PDF 5678 KB)
