# Supplementary material for: Digital Intervention Components for Preventing and Reducing Substance Use in Adolescents and Emerging Adults: A Comprehensive Scoping Review
Source: J Prev (2022). 2026 Mar 20;47(3):429–65. doi: 10.1007/s10935-025-00894-3 (PMC13283198; doi:10.1007/s10935-025-00894-3)
Supplement: Supplementary file 1 — Supplementary file1 (PDF 5678 KB) [file 10935_2025_894_MOESM1_ESM.pdf]

Supplementary file S1

Preferred Reporting Items for Systematic reviews and Meta-Analyses extension for Scoping Reviews (PRISMA-ScR) Checklist

SECTION	ITEM	PRISMA-ScR CHECKLIST ITEM	REPORTED ON PAGE #
**TITLE**			
Title	1	Identify the report as a scoping review.	Click here to enter text.
**ABSTRACT**			
Structured summary	2	Provide a structured summary that includes (as applicable): background, objectives, eligibility criteria, sources of evidence, charting methods, results, and conclusions that relate to the review questions and objectives.	Click here to enter text.
**INTRODUCTION**			
Rationale	3	Describe the rationale for the review in the context of what is already known. Explain why the review questions/objectives lend themselves to a scoping review approach.	Click here to enter text.
Objectives	4	Provide an explicit statement of the questions and objectives being addressed with reference to their key elements (e.g., population or participants, concepts, and context) or other relevant key elements used to conceptualize the review questions and/or objectives.	Click here to enter text.
**METHODS**			
Protocol and registration	5	Indicate whether a review protocol exists; state if and where it can be accessed (e.g., a Web address); and if available, provide registration information, including the registration number.	Click here to enter text.
Eligibility criteria	6	Specify characteristics of the sources of evidence used as eligibility criteria (e.g., years considered, language, and publication status), and provide a rationale.	Click here to enter text.
Information sources*	7	Describe all information sources in the search (e.g., databases with dates of coverage and contact with authors to identify additional sources), as well as the date the most recent search was executed.	Click here to enter text.
Search	8	Present the full electronic search strategy for at least 1 database, including any limits used, such that it could be repeated.	Click here to enter text.
Selection of sources of evidence†	9	State the process for selecting sources of evidence (i.e., screening and eligibility) included in the scoping review.	Click here to enter text.
Data charting process‡	10	Describe the methods of charting data from the included sources of evidence (e.g., calibrated forms or forms that have been tested by the team before their use, and whether data charting was done independently or in duplicate) and any processes for obtaining and confirming data from investigators.	Click here to enter text.
Data items	11	List and define all variables for which data were sought and any assumptions and simplifications made.	Click here to enter text.

St. Michael's
Inspired Care.
Inspiring Science.

SECTION	ITEM	PRISMA-ScR CHECKLIST ITEM	REPORTED ON PAGE #
Critical appraisal of individual sources of evidence§	12	If done, provide a rationale for conducting a critical appraisal of included sources of evidence; describe the methods used and how this information was used in any data synthesis (if appropriate).	Click here to enter text.
Synthesis of results	13	Describe the methods of handling and summarizing the data that were charted.	Click here to enter text.
**RESULTS**			
Selection of sources of evidence	14	Give numbers of sources of evidence screened, assessed for eligibility, and included in the review, with reasons for exclusions at each stage, ideally using a flow diagram.	Click here to enter text.
Characteristics of sources of evidence	15	For each source of evidence, present characteristics for which data were charted and provide the citations.	Click here to enter text.
Critical appraisal within sources of evidence	16	If done, present data on critical appraisal of included sources of evidence (see item 12).	Click here to enter text.
Results of individual sources of evidence	17	For each included source of evidence, present the relevant data that were charted that relate to the review questions and objectives.	Click here to enter text.
Synthesis of results	18	Summarize and/or present the charting results as they relate to the review questions and objectives.	Click here to enter text.
**DISCUSSION**			
Summary of evidence	19	Summarize the main results (including an overview of concepts, themes, and types of evidence available), link to the review questions and objectives, and consider the relevance to key groups.	Click here to enter text.
Limitations	20	Discuss the limitations of the scoping review process.	Click here to enter text.
Conclusions	21	Provide a general interpretation of the results with respect to the review questions and objectives, as well as potential implications and/or next steps.	Click here to enter text.
**FUNDING**			
Funding	22	Describe sources of funding for the included sources of evidence, as well as sources of funding for the scoping review. Describe the role of the funders of the scoping review.	Click here to enter text.

JBI = Joanna Briggs Institute; PRISMA-ScR = Preferred Reporting Items for Systematic reviews and Meta-Analyses extension for Scoping Reviews.

* Where *sources of evidence* (see second footnote) are compiled from, such as bibliographic databases, social media platforms, and Web sites.

† A more inclusive/heterogeneous term used to account for the different types of evidence or data sources (e.g., quantitative and/or qualitative research, expert opinion, and policy documents) that may be eligible in a scoping review as opposed to only studies. This is not to be confused with *information sources* (see first footnote).

‡ The frameworks by Arksey and O'Malley (6) and Levac and colleagues (7) and the JBI guidance (4, 5) refer to the process of data extraction in a scoping review as data charting.

§ The process of systematically examining research evidence to assess its validity, results, and relevance before using it to inform a decision. This term is used for items 12 and 19 instead of "risk of bias" (which is more applicable to systematic reviews of interventions) to include and acknowledge the various sources of evidence that may be used in a scoping review (e.g., quantitative and/or qualitative research, expert opinion, and policy document).

From: Tricco AC, Lillie E, Zarin W, O'Brien KK, Colquhoun H, Levac D, et al. PRISMA Extension for Scoping Reviews (PRISMAScR): Checklist and Explanation. Ann Intern Med. 2018;169:467–473. doi: 10.7326/M18-0850.

Supplementary file S2

Search History: CINAHL – 26 May 2023 -> 1,320

Friday, May 26, 2023 2:19:20 PM

#	Query	Limiters/Expanders	Last Run Via	Results
S13	S3 AND S6 AND S9 AND S12	Expanders - Apply equivalent subjects<br>Search modes - Boolean/Phrase	Interface - EBSCOhost Research Databases<br>Search Screen - Advanced Search<br>Database - CINAHL	1,320
S12	S10 OR S11	Expanders - Apply equivalent subjects<br>Search modes - Boolean/Phrase	Interface - EBSCOhost Research Databases<br>Search Screen - Advanced Search<br>Database - CINAHL	95,620
S11	AB (addiction or substance abuse or drug abuse) OR AB (alcoholism or alcohol dependence or alcohol abuse or alcoholic or alcohol addiction) OR AB alcohol use OR AB alcohol consumption OR AB (alcohol and other drugs) OR AB (substance use or substance abuse or drug use or drug abuse or dependence or addiction) OR AB drinking alcohol	Limiters - Peer Reviewed; Human<br>Expanders - Apply related words; Apply equivalent subjects<br>Search modes - Boolean/Phrase	Interface - EBSCOhost Research Databases<br>Search Screen - Advanced Search<br>Database - CINAHL	85,911
S10	TI (addiction or substance abuse or drug abuse) OR TI (alcoholism or alcohol dependence or alcohol abuse or alcoholic or alcohol addiction) OR TI alcohol use OR TI alcohol consumption OR TI (alcohol and other drugs) OR TI (substance use or substance abuse or drug use or drug abuse or dependence or addiction) OR TI drinking alcohol	Limiters - Peer Reviewed; Human<br>Expanders - Apply related words; Apply equivalent subjects<br>Search modes - Boolean/Phrase	Interface - EBSCOhost Research Databases<br>Search Screen - Advanced Search<br>Database - CINAHL	32,210
S9	S7 OR S8	Expanders - Apply equivalent subjects<br>Search modes - Boolean/Phrase	Interface - EBSCOhost Research Databases<br>Search Screen - Advanced Search<br>Database - CINAHL	766,028

S8	AB (interventions or strategies or best practices) OR AB (prevention or reduction or minimize) OR AB prevention programs OR AB (reduce or decrease or minimize or prevent)	Limiters - Peer Reviewed; Human<br>Expanders - Apply related words; Apply equivalent subjects<br>Search modes - Boolean/Phrase	Interface - EBSCOhost Research Databases<br>Search Screen - Advanced Search<br>Database - CINAHL	732,419
S7	TI (interventions or strategies or best practices) OR TI (prevention or reduction or minimize) OR TI prevention programs OR TI (reduce or decrease or minimize or prevent)	Limiters - Peer Reviewed; Human<br>Expanders - Apply related words; Apply equivalent subjects<br>Search modes - Boolean/Phrase	Interface - EBSCOhost Research Databases<br>Search Screen - Advanced Search<br>Database - CINAHL	137,082
S6	S4 OR S5	Expanders - Apply equivalent subjects<br>Search modes - Boolean/Phrase	Interface - EBSCOhost Research Databases<br>Search Screen - Advanced Search<br>Database - CINAHL	92,238
S5	AB app NOT AB amyloid precursor protein OR AB app based intervention OR AB app based digital OR AB virtual OR AB (web-based interventions' or 'e-health' or 'internet-based interventions) OR AB computer-based intervention OR AB digital technology OR AB (digital media or social media or the internet or online) OR AB (mobile phones or smart phones or cell phones) OR AB multimedia OR AB (text messages or text messaging service or text messaging or sms)	Limiters - Peer Reviewed; Human<br>Expanders - Apply related words; Apply equivalent subjects<br>Search modes - Boolean/Phrase	Interface - EBSCOhost Research Databases<br>Search Screen - Advanced Search<br>Database - CINAHL	86,382
S4	TI app NOT TI amyloid precursor protein OR TI app based intervention OR TI app based digital OR TI virtual OR TI (web-based interventions' or 'e-health' or 'internet-based interventions) OR TI computer-based intervention OR TI digital technology OR TI (digital media or social media or the internet or online) OR TI (mobile phones or smart phones or cell phones) OR TI multimedia OR TI (text messages or text messaging service or text messaging or sms)	Limiters - Peer Reviewed; Human<br>Expanders - Apply related words; Apply equivalent subjects<br>Search modes - Boolean/Phrase	Interface - EBSCOhost Research Databases<br>Search Screen - Advanced Search<br>Database - CINAHL	28,382

S3	S1 OR S2	Expanders - Apply equivalent subjects<br>Search modes - Boolean/Phrase	Interface - EBSCOhost Research Databases<br>Search Screen - Advanced Search<br>Database - CINAHL	493,266
S2	AB (adolescents or teenagers or young adults or teen or youth) OR AB (pupils or students) OR AB youngster OR AB young people OR AB (young men or young males or boys or male youth and male teenagers or male adolescents) OR AB (young women or young females or teenage girl or adolescent girls) OR AB (secondary school or high school or secondary education or junior high or middle school) OR AB young population	Limiters - Peer Reviewed; Human<br>Expanders - Apply related words; Apply equivalent subjects<br>Search modes - Boolean/Phrase	Interface - EBSCOhost Research Databases<br>Search Screen - Advanced Search<br>Database - CINAHL	436,424
S1	TI (adolescents or teenagers or young adults or teen or youth) OR TI (pupils or students) OR TI youngster OR TI young people OR TI (young men or young males or boys or male youth and male teenagers or male adolescents) OR TI (young women or young females or teenage girl or adolescent girls) OR TI (secondary school or high school or secondary education or junior high or middle school) OR TI young population	Limiters - Peer Reviewed; Human<br>Expanders - Apply related words; Apply equivalent subjects<br>Search modes - Boolean/Phrase	Interface - EBSCOhost Research Databases<br>Search Screen - Advanced Search<br>Database - CINAHL	288,613

Supplementary file S3

Search history: PsychInfo - 27 May 2023 -> 2,009

Datenbank:

APA PsycInfo <1806 to May Week 4 2023>

#	Abfrage	Ergebnisse aus 27 Mai 2023
1	adolescen*.ab. or adolescen*.ti. or juvenil*.ab. or juvenil*.ti. or pupil*.ab. or pupil*.ti. or teen*.ab. or teen*.ti. or youth*.ab. or youth*.ti. or youngster*.ab. or youngster*.ti. or young people.ab. or young people.ti. or young individual*.ab. or young individual*.ti. or young adult*.ab. or young adult*.ti. or young population*.ab. or young population*.ti. or young man.ab. or young man.ti. or young men.ab. or young men.ti. or young woman.ab. or young woman.ti. or young women.ab. or young women.ti. or secondary school.ab. or ssecondary school.ti. or high school.ab. or high school.ti. or student.ab. or student.ti. or adolescent.mh. or adolescents.mh. or adolescence.mh. or teens.mh. or teen.mh. or teenagers.mh. or teenager.mh. or youths.mh. or youth.mh.	842,955
2	limit 1 to human	789,986
3	limit 2 to (peer reviewed journal and human)	607,949
4	(app.ab. or app.ti. or app-based.ab. or app-based.ti. or computer*.ab. or computer*.ti. or digital*.ab. or digital*.ti. or internet*.ab. or internet*.ti. or mobile phone*.ab. or mobile phone*.ti. or multimedia.ab. or multimedia.ti. or online*.ab. or online*.ti. or social media.ab. or social media.ti. or virtual*.ab. or virtual*.ti. or web.ab. or web.ti. or text message.ab. or text message.ti. or multi media.ab. or multi media.ti.) not amyloid precursor protein.ab. not amyloid precursor protein.ti.	340,566
5	limit 4 to (peer reviewed journal and human)	236,792
6	intervent*.ab. or intervent*.ti. or prevent*.ab. or prevent*.ti. or reduct*.ab. or reduct*.ti. or reduce*.ab. or reduce*.ti. or consumption reduction.ab. or consumtion reduction.ti.	984,810
7	limit 6 to (peer reviewed journal and human)	669,938
8	addict*.ab. or addict*.ti. or alcohol*.ab. or alcohol*.ti. or AOD.ab. or AOD.ti. or SUD.ab. or SUD.ti. or alcohol abuse.mh. or alcohol drinking.mh. or substance abuse.mh. or SUD.mh.	192,818
9	limit 8 to (peer reviewed journal and human)	139,216
10	3 and 5 and 7 and 9	2,009

Supplementary file S4

Search history: PubMed - 26 May 2023 -> 4,040

Search number,Query,Sort By,Filters,Search Details,Results,Time

"5,((((#4) AND (#1)) AND (#2)) AND (#3)) AND (#4),,Humans,""((""""addict*""""[Title/Abstract] OR """"alcohol*""""[Title/Abstract] OR """"AOD""""[Title/Abstract] OR """"SUD""""[Title/Abstract] OR """"substance use disorder""""[Title/Abstract] OR """"alcoholism""""[MeSH Terms] OR """"alcohol drinking""""[MeSH Terms] OR """"substance related disorders""""[MeSH Terms] OR """"substance related disorders""""[MeSH Terms] OR """"substance related disorders""""[MeSH Terms]) AND """"humans""""[MeSH Terms] AND ((""""adolescen*""""[Title/Abstract] OR """"juvenil*""""[Title/Abstract] OR """"pupil*""""[Title/Abstract] OR """"teen*""""[Title/Abstract] OR """"youth*""""[Title/Abstract] OR """"youngster*""""[Title/Abstract] OR """"young people""""[Title/Abstract] OR """"young individual*""""[Title/Abstract] OR """"young adult*""""[Title/Abstract] OR """"young population""""[Title/Abstract] OR """"young man""""[Title/Abstract] OR """"young men""""[Title/Abstract] OR """"young woman""""[Title/Abstract] OR """"young women""""[Title/Abstract] OR """"secondary school""""[Title/Abstract] OR """"high school""""[Title/Abstract] OR """"student*""""[Title/Abstract] OR """"adolescent""""[MeSH Terms] OR """"adolescent""""[MeSH Terms] OR """"adolescent""""[MeSH Terms] OR """"adolescent""""[MeSH Terms] OR """"adolescent""""[MeSH Terms] OR """"adolescent""""[MeSH Terms] OR """"adolescent""""[MeSH Terms] OR """"adolescent""""[MeSH Terms] OR """"adolescent""""[MeSH Terms]) AND """"humans""""[MeSH Terms]) AND (((""""app""""[Title/Abstract] NOT """"amyloid precursor protein""""[Title/Abstract]) OR """"computer""""[Title/Abstract] OR """"digital*""""[Title/Abstract] OR """"internet*""""[Title/Abstract] OR """"mobile phone*""""[Title/Abstract] OR """"multimedia""""[Title/Abstract] OR """"multi media""""[Title/Abstract] OR """"online*""""[Title/Abstract] OR """"social media""""[Title/Abstract] OR """"virtual*""""[Title/Abstract] OR """"web""""[Title/Abstract] OR """"text message""""[Title/Abstract]) AND """"humans""""[MeSH Terms]) AND ((""""intervent*""""[Title/Abstract] OR """"prevent*""""[Title/Abstract] OR """"reduct*""""[Title/Abstract] OR """"reduce*""""[Title/Abstract] OR """"consumption reduction""""[Title/Abstract]) AND """"humans""""[MeSH Terms]) AND ((""""addict*""""[Title/Abstract] OR """"alcohol*""""[Title/Abstract] OR """"AOD""""[Title/Abstract] OR """"SUD""""[Title/Abstract] OR """"substance use disorder""""[Title/Abstract] OR """"alcoholism""""[MeSH Terms] OR """"alcohol drinking""""[MeSH Terms] OR """"substance related disorders""""[MeSH Terms] OR """"substance related disorders""""[MeSH Terms] OR """"substance related disorders""""[MeSH Terms]) AND """"humans""""[MeSH Terms])) AND (humans[Filter])"",""4,040"",07:32:33"

"4,""(((((((((addict*[Title/Abstract]) OR (alcohol*[Title/Abstract])) OR (AOD[Title/Abstract])) OR (SUD[Title/Abstract])) OR (substance use disorder[Title/Abstract])) OR (alcohol abuse[MeSH Terms])) OR (alcohol drinking[MeSH Terms]))) OR (addiction, substance[MeSH Terms])) OR (substance use disorder[MeSH Terms])) OR (substance use disorders[MeSH Terms])"",,Humans,""(""""addict*""""[Title/Abstract] OR """"alcohol*""""[Title/Abstract] OR """"AOD""""[Title/Abstract] OR """"SUD""""[Title/Abstract] OR """"substance use disorder""""[Title/Abstract] OR """"alcoholism""""[MeSH Terms] OR """"alcohol drinking""""[MeSH

Terms] OR """"substance related disorders""""[MeSH Terms] OR """"substance related disorders""""[MeSH Terms] OR """"substance related disorders""""[MeSH Terms]) AND (humans[Filter])"",""470,705"",07:31:14"

"3,((((intervent*[Title/Abstract]) OR (prevent*[Title/Abstract])) OR (reduct*[Title/Abstract])) OR (reduce*[Title/Abstract])) OR (consumption reduction[Title/Abstract]),,Humans,""(""""intervent*""""[Title/Abstract] OR """"prevent*""""[Title/Abstract] OR """"reduct*""""[Title/Abstract] OR """"reduce*""""[Title/Abstract] OR """"consumption reduction""""[Title/Abstract]) AND (humans[Filter])"",""3,634,072"",07:28:27"

"2,((((((((((app[Title/Abstract]) NOT (amyloid precursor protein[Title/Abstract])) OR (computer[Title/Abstract])) OR (digital*[Title/Abstract])) OR (internet*[Title/Abstract])) OR (mobile phone*[Title/Abstract])) OR (multimedia[Title/Abstract])) OR (multi media[Title/Abstract])) OR (online*[Title/Abstract])) OR (social media[Title/Abstract])) OR (virtual*[Title/Abstract])) OR (web[Title/Abstract])) OR (text message[Title/Abstract]),,Humans,""((""""app""""[Title/Abstract] NOT """"amyloid precursor protein""""[Title/Abstract]) OR """"computer""""[Title/Abstract] OR """"digital*""""[Title/Abstract] OR """"internet*""""[Title/Abstract] OR """"mobile phone*""""[Title/Abstract] OR """"multimedia""""[Title/Abstract] OR """"multi media""""[Title/Abstract] OR """"online*""""[Title/Abstract] OR """"social media""""[Title/Abstract] OR """"virtual*""""[Title/Abstract] OR """"web""""[Title/Abstract] OR """"text message""""[Title/Abstract]) AND (humans[Filter])"",""637,848"",07:27:38"

"1,((((((((((((((((((((((adolescen*[Title/Abstract]) OR (juvenil*[Title/Abstract])) OR (pupil*[Title/Abstract])) OR (teen*[Title/Abstract])) OR (youth*[Title/Abstract])) OR (youngster*[Title/Abstract])) OR (young people[Title/Abstract])) OR (young individual*[Title/Abstract])) OR (young adult*[Title/Abstract])) OR (young population[Title/Abstract])) OR (young man[Title/Abstract])) OR (young men[Title/Abstract])) OR (young woman[Title/Abstract])) OR (young women[Title/Abstract])) OR (secondary school[Title/Abstract])) OR (high school[Title/Abstract])) OR (student*[Title/Abstract])) OR (adolescence[MeSH Terms])) OR (adolescent[MeSH Terms])) OR (adolescents[MeSH Terms])) OR (teen[MeSH Terms])) OR (teens[MeSH Terms])) OR (teenager[MeSH Terms])) OR (teenagers[MeSH Terms])) OR (youth[MeSH Terms])) OR (youths[MeSH Terms]),,Humans,""(""""adolescen*""""[Title/Abstract] OR """"juvenil*""""[Title/Abstract] OR """"pupil*""""[Title/Abstract] OR """"teen*""""[Title/Abstract] OR """"youth*""""[Title/Abstract] OR """"youngster*""""[Title/Abstract] OR """"young people""""[Title/Abstract] OR """"young individual*""""[Title/Abstract] OR """"young adult*""""[Title/Abstract] OR """"young population""""[Title/Abstract] OR """"young man""""[Title/Abstract] OR """"young men""""[Title/Abstract] OR """"young woman""""[Title/Abstract] OR """"young women""""[Title/Abstract] OR """"secondary school""""[Title/Abstract] OR """"high school""""[Title/Abstract] OR """"student*""""[Title/Abstract] OR """"adolescent""""[MeSH Terms] OR """"adolescent""""[MeSH Terms] OR """"adolescent""""[MeSH Terms] OR """"adolescent""""[MeSH Terms] OR """"adolescent""""[MeSH Terms] OR """"adolescent""""[MeSH Terms] OR """"adolescent""""[MeSH Terms] OR """"adolescent""""[MeSH Terms] OR """"adolescent""""[MeSH Terms]) AND (humans[Filter])"",""2,578,933"",06:15:33"

Supplementary file S5

Search history: Web of Science – 27 May 2023 -> 113

Database: Web of Science Core Collection

Entitlements:
- WOS.IC: 1993 to 2023
- WOS.CCR: 1985 to 2023
- WOS.SCI: 1900 to 2023
- WOS.AHCI: 1975 to 2023
- WOS.BHCI: 2005 to 2023
- WOS.BSCI: 2005 to 2023
- WOS.ESCI: 2005 to 2023
- WOS.ISTP: 1990 to 2023
- WOS.SSCI: 1900 to 2023
- WOS.ISSHP: 1990 to 2023

Searches:

1: ((((((((((((((((((((((((((((((AB=(adolescents)) OR AB=(juvenile)) OR AB=(pupil)) OR AB=(teen)) OR AB=(youths)) OR AB=(youngster)) OR AB=(young people)) OR AB=(young individual)) OR AB=(young adult)) OR AB=(young population)) OR AB=(young man)) OR AB=(young men)) OR AB=(young woman)) OR AB=(young women)) OR AB=("secondary school")) OR AB=("high school")) OR AB=(student)) OR TI=(adolescents)) OR TI=(juvenile)) OR TI=(pupil)) OR TI=(teen)) OR TI=(youths)) OR TI=(youngster)) OR TI=(young people)) OR TI=(young individual)) OR TI=(young adult)) OR TI=(young population)) OR TI=(young man)) OR TI=(young men)) OR TI=(young woman)) AND TI=(young women)) OR TI=("secondary school")) OR TI=("high school") Date Run: Sat May 27 2023 13:50:13 GMT+0200 (Mitteleuropäische Sommerzeit) Results: 67558

2: (((((((((((((((((((((((AB=(app)) OR AB=(computer)) OR AB=(digital)) OR AB=(internet)) OR AB=(mobile phone)) OR AB=(multimedia)) OR AB=("multi media")) OR AB=(online)) OR AB=("social media")) OR AB=(virtual)) OR AB=(web)) OR AB=("text message")) NOT AB=("amyloid precursor protein")) OR TI=(app)) OR TI=(computer)) OR TI=(digital)) OR TI=(internet)) OR TI=(mobile phone)) OR TI=(multimedia)) OR TI=("multi media")) OR TI=(online)) OR TI=("social media")) OR TI=(virtual)) OR TI=(web)) OR TI=("text message")) NOT TI=("amyloid precursor protein")

Date Run: Sat May 27 2023 13:59:31 GMT+0200 (Mitteleuropäische Sommerzeit)

Results: 3188550

3: (((((((((AB=(intervention)) OR AB=(prevention)) OR AB=(reduction)) OR AB=(reduce)) OR AB=("consumption reduction")) OR TI=(intervention)) OR TI=(prevention)) OR TI=(reduction)) OR TI=(reduce)) OR TI=("consumption reduction") Date Run: Sat May 27 2023 14:02:08 GMT+0200 (Mitteleuropäische Sommerzeit) Results: 8733561

4: (((((((((AB=(addiction)) OR AB=(alcohol)) OR AB=(AOD)) OR AB=(SUD)) OR AB=("substance use disorder")) OR TI=(addiction)) OR TI=(alcohol)) OR TI=(AOD)) OR TI=(SUD)) OR TI=("substance use disorder") Date Run: Sat May 27 2023 14:12:54 GMT+0200 (Mitteleuropäische Sommerzeit) Results: 589888

5: #4 AND #3 AND #2 AND #1
GMT+0200 (Mitteleuropäische Sommerzeit)

Date Run: Sat May 27 2023 14:13:21
Results: 113

Supplementary file S6_Characteristics of included studies

	Intervention	Publication	Country	Sample size*		Follow-up
				Baseline	Latest follow-up	Latest (months)
**A Adolescents**						
**1**	Alc-Check	Haug et al., 2013	UK	364	280	3
**2**	Alcohol Alert / Alerta Alcohol	Jander et al., 2016	NL	2649	824	4
		Martinez-Montilla et al., 2020	US	1247	612	4
		Vargas-Martinez et al., 2019	US	1247	612	4
**3**	Click City®: Alcohol	Gordon et al., 2017	US	2855	2609	0.25
**4**	Climate Schools	Newton et al., 2022a	US	2190	1038	84
		Vogl et al., 2009	US	1466	1016	12
**5**	eCHUG (electronic version of Check-up to go)	Doumas et al., 2019	US	125	105	6
		Doumas et al., 2020a	US	311	283	1
		Doumas et al., 2020b	US	135	123	1
		Doumas et al., 2021	US	311	247	6
**6**	HSD (Healthy School and Drugs)	Koning et al., 2011	US	3490	2937	34
**7**	kiR (keepin' it REAL)	Shin et al., 2018	US	1464	n.r.	1
**8**	MobileCoach Alcohol	Haug et al., 2017b	US	1041	966	6
		Haug et al., 2020	US	633	136	0
		Haug et al., 2023	US	954	274	0
**9**	PALMSS (peer alcohol knowledge, low-risk drinking, media-influence, social drinking, self-efficacy)	Hongthon et al., 2016	UK	150	150	6
**10**	Smart Choices 4 Teens	Byrnes et al., 2019	US	411 dyades	315 teens, 364 parents	12
**11**	SPORT	Mathews et al., 2007	US	217	217	0
**12**	VR *FestLab* (virtual reality FestLab)	Guldager et al., 2022	US	378	214	1.5
**13**	WDYD (What do you drink?)	Voogt et al., 2013	US	609	329	6
**14**	*No name*	Ho et al., 2021	US	7792	6700	3
**15**	*No name*	Knight et al., 2018	US	2096 (US) & 588 (CZ)	1545 (US) & 532 (CZ)	12
**16**	*No name*	Russell et al., 2017	US	762	762	0
**17**	*No name*	Schinke et al., 2009b	US	202 pairs	199 pairs	2
**18**	C2Q-Teen (Craving to Quit-Teen)	Pbert et al., 2020	US	146	146 (ggf imputations)	6
**19**	QuitSTART smoking app	Pbert et al., 2020	US	146	146 (ggf imputations)	6
**20**	*No name*	Kong et al., 2017	US	15	12	1
**21**	*No name*	Mason et al., 2015	US	72	22	6
**22**	*No name*	Shi et al., 2013	UK	179	122	0

**23**	Invite Only VR (virtual reality)	Weser et al., 2021	US	287	201	6
**24**	smokeSCREEN	Bteddini et al., 2023	US	82 (quant)	60 (quant)	4.25
**25**	xkpts.com ((reader: “perquèpetes.com”, translation: “why joints?”)	Ariza et al., 2013	US	4848	3191	15
**26**	P4T (POP4Teens) vs JTT (JustThinkTwice)	Marsch et al., 2021	AU	405	283	6
**27**	Elm City Story (PlayForward)	Montanaro et al., 2015	NZ	166 in intervention group	144	6
**28**	REAL media	Greene et al., 2020	US	639	n.r. (639 intent-to-treat)	3
**29**	*No name*	Fang & Schinke, 2013	US	108	93 pairs	24
**30**	E-MOVO (Dutch for Electronic Monitoring and Health Promotion)	Crutzen et al., 2008	US	35104	33644	2
**31**	Health4Life	Champion et al., 2023	NL	6640	5015	24
**32**	Climate Schools	Champion et al., 2016	FR	1103	880	0
		Newton et al., 2022b	US	6386	5343	12
**33**	*No name*	Gryczynski et al., 2021	US	350	277	6
**34**	*No name*	Knight et al., 2019	US	869	710	12
**35**	Healthy School and Drugs	Malmberg et al., 2014	US	3542	2340	32
**36**	¿Qu é pasa si te pasas? (What happens if you go to far?)	Fuentes et al., 2023	CH	359	356	0
**37**	ready4life	Haug et al., 2022	US	1351	962	6
**38**	SmartCoach	Paz Castro et al., 2022	US	1473	1232	18
**39**	RealTeen	Schwinn et al., 2019	US	788	727	36
		Schwinn et al., 2010	US	236	215	6
**40**	Vamos	Schwinn et al., 2021	US	644	572	36
**41**	*No name (family interaction)*	Schinke et al., 2009a	US	916 pairs	828 pairs	24
		Schinke et al., 2011	US	546 pairs	546 pairs	0
**42**	*No name*	Schwinn et al., 2015	IN	236	200	3
**43**	E-health4Uth	Bannink et al., 2014	UK	1702	1256	4
**44**	Your Decision Counts TM	Evers et al., 2012	US	1590	n.r. (last observation carried forward)	14
**45**	WISEteens	Arnaud et al., 2016	US	1449	211	3
**46**	Girls and Stress	Schinke et al., 2005	US	91	91	0.5
**47**	*No name*	Williams et al., 2005	US	193	123	0
**48**	Pure Rush	Stapinski et al., 2018	US	281	281	0
**49**	Climate Schools Psychostimulant & Cannabis Modlue	Vogl et al., 2014	DE	1734	973	10
**50**	Refuse to Use	Duncan et al., 2020	US	65	65	0
**51**	Climate Schools: Ecstasy and Emerging Drugs Module	Champion et al., 2018	AU	1126	692	24
**B Emerging adults**						
**1**	Alcohol 101 Plus™	Carey et al., 2011 (study 2)	NL	677	460	12
**2**	Alcohol 101	Barnett et al., 2007	ES	225	212	12
		Braitman et al., 2016	ES	353	115	1
		Carey et al., 2009	US	198	198	12
**3**	Alcohol Edu® for Sanctions	Carey et al., 2011 (study 1)	AU	677	460	12
**4**	Alcohol Edu® for College	Croom et al., 2009	AU	3216	1917	1.5
		Hustad et al., 2010 (A)	US	82	80	1

		Paschall et al., 2014	US	2400	n.r.	5
**5**	Alcohol-Wise	Croom et al., 2015 (study 1)	US	3022	2007	1.6
		Croom et al., 2015 (study 2)	US	2723	2027	1.6
		Gilbertson et al., 2017	NL	126	64	6
		Strohman et al., 2016	US	58	58	1
**6**	alcooquizz.ch (1)	Bertholet et al., 2015	CH	737	737	6
		Bertholet et al., 2016	CH	737	734	6
		Bertholet et al., 2018	CH	737	626	47
**7**	BASICS (Brief Alcohol Screening and Intervention for College Students)	Lee et al., 2014	TH	783	729	0.25
		Linowski et al., 2016	US	791	346	12
		Murphy et al., 2010, (study 1)	US	74	69	1
		Murphy et al., 2010 (study 2)	DK	133	118	1
		Patrick et al., 2014	NL	271	263	0
		Patrick et al., 2021	CN	891	735	0
		Saitz et al., 2007	US & CZ	2194	1398	1
**8**	BASICS (Brief Alcohol Screening and Intervention for College Students) - PNF component	LaBrie et al., 2013	US	1663	1646	12
		Larimer et al., 2021	US	1181 (USA), 2230 (Sweden)	835 (USA) & 1192 (Sweden)	12
		Moreira et al., 2012	US	2611	1050	12
		Neighbors et al., 2004	US	252	207	6
		Neighbors et al., 2006	US	214	185	2
		Neighbors et al., 2010	US	818	665	24
**9**	Campus GANDR (Gamified Alcohol Norm Discovery & Readjustment)	Boyle et al., 2017	CH	273	250 / 252	0.5
**10**	CDCU (College Drinker's Check-up)	Carey et al., 2017	US	326	251	6
		Hester et al., 2012 (study 1)	US	144	130	12
		Hester et al., 2012 (study 2)	ES	82	81	1
**11**	CIAN (Call it a Night®)	Fucito et al., 2017	US	42	38	3
**12**	College Alc	Bersamin et al., 2007	US	622	370	3
**13**	D_ARIANNA ((Digital-Alcohol Risk Alertness Notifying Network for Adolescents and Young Adults))	Carrà et al., 2016	US	590	507	0.5
**14**	DrAFT-CS (Drinking Assessment and Feedback Tool for College Students)	Wagener et al., 2012	US	152	142	2.5
		Weaver et al., 2014	NL	176	157	1
**15**	ECALC (expectancy challenge alcohol literacy curriculum)	Dunn et al., 2020 (B) 2019?	AU	134	110	1
**16**	eCHUG (electronic version of Check-up to go)	Alfonso et al., 2012	AU	173	173	3
		Braitman et al., 2022	AU	545	528	3
		Doumas et al., 2009	US	76	67	8
		Hustad et al., 2010 (B)	US	82	80	1
		Thompson et al., 2018	NL	245	143	5
		Tahaney & Palfay, 2017	MX	113	111	1
		Walters et al., 2007	CH	106	n.r. (106 imputed)	4
**17**	eentjeteveel	Fraeyman et al., 2012	CH	34	34	0
**18**	eSBI (electronic screening and brief intervention)	Kypri et al., 2013	US	1789	1415	5
		Kypri et al., 2014	CA	3422	2850 (5 outcomes); 2761 (6 outcomes)	5
		Kypri et al., 2008	US & PR	576	360	12

**19**	ETUCARE	Theurel et al., 2022	US	103	58	1
**20**	FIT (functional imagery training)	Shuai et al., 2022	US	76	52	0.5
**21**	M-PASS (Michigan Prevention and Alcohol Safety for Students)	Bingham et al., 2011	US	1137	742	3
**22**	MyStudentBody:Alcohol	Chiauzzi et al., 2005	NL	265	215	3
**23**	myPlaybook	Fearnow-Kenney et al., 2016	US	1356	1356	0
		Zamboanga et al., 2019	SE, DE, BE, CZ	2449	2449	4
**24**	PantherTRAC	Suffoletto et al., 2016	US	224	224	0
**25**	PartyWise	Schwarz et al., 2022	US	520	449	9
**26**	Ray's Night Out	Hides et al., 2018	AU	197	183	6
		Pocuca et al., 2016	AU	9	9	0
**27**	THRIVE (Tertiary Health Research Intervention via Email)	Kypri et al., 2009	US	2050	1578	6
		Leeman et al., 2016	AU	208	164	6
**60**	Unitcheck	Bewick et al., 2013		1478	644	8.5
		Marley et al., 2016		21	21	0
**29**	WDYD (What do you drink?)	Voogt et al., 2014a	US	820	658	6
**30**	Y1-CAP (Year 1 College Alcohol Profile)	Shell et al., 2019	US	3364 (1st) & 3111 (2nd)	3364 (1st) & 3111 (2nd)	24
**31**	No name	Bernstein et al., 2018	US	200	195	0
**32**	No name	Bonar et al., 2022	US	955	908	12
**33**	*No name*	Braitman et al., 2020	US	537	140	9
**34**	*No name*	Boyle et al., 2021	US	230	223	1
**35**	*No name*	Bryant et al., 2013	US	191	191	1.5
**36**	*No name*	Buckner et al., 2019	US	147	122	1
**37**	*No name*	Butler et al., 2009	US	84	84	1
**38**	*No name*	Carey et al., 2020	US	121	121	6
**39**	*No name*	Collins et al., 2014	US	724	537	12
**40**	*No name*	Doumas et al., 2011	US	135	83	1
**41**	*No name*	Ellis et al., 2017	CH	103	103	0
**42**	*No name*	Gex et al., 2023	CH	66	55	3
**43**	*No name*	Glowacki et al., 2020	CH	279	279	0
**44**	*No name*	Hagger et al., 2012	US	709	238	1
**45**	*No name*	Hendershot et al., 2010	US	200	179	1
**46**	*No name*	Kazemi et al., 2020 (study 1)	US	141	102	1.5
**47**	*No name*	Kazemi et al., 2020 (study 2)	US	238	171	1.5
**48**	*No name*	LaBrie et al., 2019	US	235	227	0.75
**49**	*No name*	Larimer et al., 2023	US	1137	1011	12
**50**	*No name*	Leary et al., 2022	US	147	124	1.25
**51**	*No name*	Leavens et al., 2020	US	268	156	1
**52**	*No name*	Lewis et al., 2007	US & SE	245	209	5
**53**	*No name*	Ma, 2022	UK	107	107	0
**54**	*No name*	Mason et al., 2014	US	18	18	1
**55**	No name	McGeary et al., 2014	US	41	41	0
**56**	*No name*	Neighbors et al., 2009	US	295	282	0.25
**57**	*No name*	Norman et al., 2018	US	2951	892	6
**58**	*No name*	Palfai et al., 2011	US	119	119	1
**59**	*No name*	Palfai et al., 2014a	US	1336	705	5

**60**	*No name*	Pilling et al., 2007	US	227	227	0
**61**	*No name*	Ridout & Cambell, 2014	US	95	95	3
**62**	*No name*	Riordan et al., 2023	US	783	704	8
**63**	*No name*	Savage et al., 2015	IT	231	75% at 22 week follow-up	5
**64**	*No name*	Schuckit et al., 2016	US	430	290	12
**65**	*No name*	Smallman et al., 2023	US	107	73	1
**66**	*No name*	Spijkerman et al., 2010	US	575	278	3
**67**	*No name*	Tello et al., 2018	US	126	122	0
**68**	*No name*	Thombs et al., 2007	US	Ca. 384	Ca. 384	?
**69**	*No name*	Walters et al., 2009	US	279	241	6
**70**	*No name*	Whitt et al., 2019	US	183	183	0
**71**	*No name*	Yang & Nan, 2019	CA	519	519	0
**72**	Mobile Coach Tobacco & Mobile Coach Tobacco +	Haug et al., 2017a	US	1471	1116	6
**73**	BASICS (Brief Alcohol Screening and Intervention for College Students - adaption for marijuana)	Lee et al., 2010	US	341	322	6
**74**	CAAT (Cannabis Approach Avoidance Training)	Karoly et al., 2019	BEL	41	37	0
**75**	eCHUG (electronic version of Check-up to go)marijuana	Elliott et al., 2014	NZ	317	317	1
		Palfai et al., 2014b	NZ	123	103	6
**76**	*No name*	Buckner et al., 2020	AU & NZ	63	63	0.5
**77**	*No name*	Walukevich-Dienst et al., 2019	FR	204	177	1
**78**	iHeLP	Braciszewski et al., 2018	AU	33	25	12
**79**	d-SBI (Digital screening and brief intervention for alcohol misuse)	Sharma et al., 2023	US	37	24	3
**80**	U@Uni:LifeGuide	Cameron et al., 2015	US	2621	1495	6
**81**	CAAT (Cannabis Approach Avoidance Training)	Jacobus et al., 2018	US	80	80	0
**82**	MiSARA (substance abuse research assistant)	Coughlin et al., 2021	US	39	39	1
**83**	myPlaybook	Rulison et al., 2022 (study 1)	US	3859	2193	1
		Rulison et al., 2022 (study 2)	US	2837	2145	1
		Rulison et al., 2022 (study 3)	AU	2193	2193	0
**84**	Meine Zeit ohne - Die Challenge	Pietsch et al., 2023	AU	4591	2861	0
**85**	Project Fitness	Moore et al., 2012	AU	200	200	0

* n.r. = not reported

Supplementary file S7_ Substances, settings & inclusion criteria

	Project / intervention	Publication	Target sub-stance*	Inclusion criteria		Setting	Mean age***
				Consumption patterns**	Other criteria		
**A Adolescents**							
**1**	Alc-Check	Haug et al., 2013	ALC	all		Vocational school	17.3
**2**	Alcohol Alert / Alerta Alcohol	Jander et al., 2016	ALC	all		School	16.3
		Martinez-Montilla et al., 2020	ALC	all		School	16.32
		Vargas-Martinez et al., 2019	ALC	all		School	16.8
**3**	Click City®: Alcohol	Gordon et al., 2017	ALC	all		School	n.r.
**4**	Climate Schools	Newton et al., 2022a	ALC	all		School	13.3
		Vogl et al., 2009	ALC	all		School	13
**5**	eCHUG (electronic version of Check-up to go)	Doumas et al., 2019	ALC	all		School	17.14
		Doumas et al., 2020a	ALC	all		School	17.15
		Doumas et al., 2020b	ALC	con		School	17.15
		Doumas et al., 2021	ALC	all		School	17.13
**6**	HSD (Healthy School and Drugs)	Koning et al., 2011	ALC	all	No manifest heavy weekly drinking at baseline	School	12.6
**7**	kiR (keepin' it REAL)	Shin et al., 2018	ALC	all		School	13.76
**8**	MobileCoach Alcohol	Haug et al., 2017b	ALC	all		School	16.1
		Haug et al., 2020	ALC	hcon		School	16.8
		Haug et al., 2023	ALC	all		School	16.1
**9**	PALMSS (peer alcohol knowledge, low-risk drinking, media-influence, social drinking, self-efficacy)	Hongthon et al., 2016	ALC	con	Low-risk consumers (AUDIT 1-7)	School	16.57

**10**	Smart Choices 4 Teens	Byrnes et al., 2019	ALC	all		Family	16.4
**11**	SPORT	Mathews et al., 2007	ALC	all		School	n.r.
**12**	VR *FestLab* (virtual reality FestLab)	Guldager et al., 2022	ALC	all		School	15.7
**13**	WDYD (What do you drink?)	Voogt et al., 2013	ALC	hcon	Low educational background	School	17.3
**14**	*No name*	Ho et al., 2021	ALC	all		School	13.3
**15**	*No name*	Knight et al., 2018	ALC	all	Patients presenting for routine care	Primary care	USA: 15.8; CZ: 15.0
**16**	*No name*	Russell et al., 2017	ALC	all		General Population	n.r. (14-17)
**17**	*No name*	Schinke et al., 2009b	ALC	all	Girl and mother-dyades	Family	12.2
**18**	C2Q-Teen (Craving to Quit-Teen)	Pbert et al., 2020	TOB	con	Interest in quitting	School	16.9
**19**	QuitSTART smoking app	Pbert et al., 2020	TOB	con	Interest in quitting	School	16.9
**20**	*No name*	Kong et al., 2017	TOB	con		General population (recruitment via high schools)	16.47
**21**	*No name*	Mason et al., 2015	TOB	con		General population	16.4
**22**	*No name*	Shi et al., 2013	TOB	con		School	17.6
**23**	Invite Only VR (virtual reality)	Weser et al., 2021	eTOB	all		School	12.45
**24**	smokeSCREEN	Bteddini et al., 2023	eTOB	all		General population	13.86
**25**	xkpts.com ((reader: “perquèpetes.com”, translation: “why joints?”)	Ariza et al., 2013	CAN	all		School	14.44
**26**	P4T (POP4Teens) vs JTT (JustThinkTwice)	Marsch et al., 2021	Prescription opioids	all		General population	15.8
**27**	Elm City Story (PlayForward)	Montanaro et al., 2015	DU	all		General population	12.95

**28**	REAL media	Greene et al., 2020	DU	all		Community organisations	14.71
**29**	*No name*	Fang & Schinke, 2013	DU	all	Asian-american adolescent girls & mothers	Family	13.1
**30**	E-MOVO (Dutch for Electronic Monitoring and Health Promotion)	Crutzen et al., 2008	ALC, TOB	all		School	n.r.
**31**	Health4Life	Champion et al., 2023	ALC, TOB	all		School	n.r.
**32**	Climate Schools	Champion et al., 2016	ALC, CAN	all		School	13.25
		Newton et al., 2022b	ALC, CAN	all		School	13.5
**33**	*No name*	Gryczynski et al., 2021	ALC, CAN	hcon	No other drugs consumed; school-based health care patients	School	16.3
**34**	*No name*	Knight et al., 2019	ALC, CAN	con	Patients who presented for annual preventive health visits; not medically or emotionally instable at baseline;	Primary care	16.4
**35**	Healthy School and Drugs	Malmberg et al., 2014	ALC, TOB, CAN	con		School	13.01
**36**	¿Qu é pasa si te pasas? (What happens if you go to far?)	Fuentes et al., 2023	ALC, TOB, CAN	con		School	15
**37**	ready4life	Haug et al., 2022	ALC, TOB, CAN	con	Apprentices	Vocational school	17.3
**38**	SmartCoach	Paz Castro et al., 2022	ALC, TOB, CAN	con		School	15.4
**39**	RealTeen	Schwinn et al., 2019	ALC, TOB, eTOB, CAN, MET, ECT, cocaine, inhalants, poly DU	con	Girls only	General population	15.91
		Schwinn et al., 2010	ALC, TOB, CAN, MET, ECT, cocaine, inhalants, poly DU	con	Girls only	General Population	14
**40**	Vamos	Schwinn et al., 2021	ALC, TOB, CAN, poly DU	con	Hispanic youths only	General population	14.1
**41**	*No name (family interaction)*	Schinke et al., 2009a	ALC, TOB, CAN, PD	con	Girl and mother-dyades	Family	12.76

		Schinke et al., 2011	ALC, TOB, CAN, PD	con	Black & hispanic girl and mother-dyades	Family	12.75
**42**	*No name*	Schwinn et al., 2015	ALC, TOB, CAN, DU	con	Identification as lgbtq	General population	16.08
**43**	E-health4Uth	Bannink et al., 2014	ALC, TOB, CAN, DU	con		School	15.9
**44**	Your Decision Counts TM	Evers et al., 2012	ALC, TOB, CAN, polyDU	con		School	n.r.
**45**	WISEteens	Arnaud et al., 2016	ALC, polyDU	hcon		General population	16.8
**46**	Girls and Stress	Schinke et al., 2005	ALC, TOB, CAN, crack, heroine, DU	all	Girls only	School	n.r.
**47**	*No name*	Williams et al., 2005	ALC, TOB, CAN, inhalants	all		School	n.r.
**48**	Pure Rush	Stapinski et al., 2018	CAN, MET, hallucinogenes	all		School	14.3
**49**	Climate Schools Psychostimulant & Cannabis Modlue	Vogl et al., 2014	CAN, psychostimulants	all		School	15.44
**50**	Refuse to Use	Duncan et al., 2020	CAN, DU	all	Multi-cultural population (non-Hispanic Caucasian, African-American, Hispanic)	School	15.2
**51**	Climate Schools: Ecstasy and Emerging Drugs Module	Champion et al., 2018	Ecstasy & emerging drugs	all		School	14.9
**B Emerging adults**							
**1**	Alcohol 101 Plus™	Carey et al., 2011	ALC	con		University	19
**2**	Alcohol 101	Barnett et al., 2007	ALC	con		University	18.8
		Braitman et al., 2016	ALC	all		University	n.r.
		Carey et al., 2009	ALC	con		University	19.17
**3**	Alcohol Edu® for Sanctions	Carey et al., 2011	ALC	con		University	19
**4**	Alcohol Edu® for College	Croom et al., 2009	ALC	all		University	n.r. (freshmen)
		Hustad et al., 2010 (A)	ALC	all		University	18.1
		Paschall et al., 2014	ALC	all		University	18.7

**5**	Alcohol-Wise	Croom et al., 2015 (study 1)	ALC	all		University	n.r.
		Croom et al., 2015 (study 2)	ALC	all		University	n.r.
		Gilbertson et al., 2017	ALC	all		University	n.r.
		Strohman et al., 2016	ALC	all		University	20
**6**	alcooquizz.ch (1)	Bertholet et al., 2015	ALC	hcon	Participants from C-SURF project	General population	20.75
		Bertholet et al., 2016	ALC	hcon	Participants from C-SURF project	General population	20.8
		Bertholet et al., 2018	ALC	hcon	Participants from C-SURF project	General population	20.8
**7**	BASICS (Brief Alcohol Screening and Intervention for College Students)	Lee et al., 2014	ALC	hcon	Intention to go on spring-break trip & drink heavily	University	20.5
		Linowski et al., 2016	ALC	hcon		University	n.r.
		Murphy et al., 2010, (study 1)	ALC	hcon		University	21.2
		Murphy et al., 2010 (study 2)	ALC	hcon		University	18.6
		Patrick et al., 2014	ALC	all	Intention to go on spring-break trip	University	n.r.
		Patrick et al., 2021	ALC	all		University	18.1
		Saitz et al., 2007	ALC	hcon		University	n.r.
**8**	BASICS (Brief Alcohol Screening and Intervention for College Students) - PNF component	LaBrie et al., 2013	ALC	hcon	Caucasian or asien origin only included	University	19.92
		Larimer et al., 2021	ALC	all		University	17,58 (USA), 17,83 (SE)
		Moreira et al., 2012	ALC	all		University	n.r.
		Neighbors et al., 2004	ALC	hcon		University	18,5
		Neighbors et al., 2006	ALC	hcon		University	19.67
		Neighbors et al., 2010	ALC	hcon		University	n.r.
**9**	Campus GANDR (Gamified Alcohol Norm Discovery & Readjustment)	Boyle et al., 2017	ALC	all	Psychology students	University	n.r.
**10**		Carey et al., 2017	ALC	hcon	Living off-campus	University	20.97

	CDCU (College Drinker's Check-up)	Hester et al., 2012 (study 1)	ALC	hcon		University	n.r.
		Hester et al., 2012 (study 2)	ALC	hcon		University	n.r.
**11**	CIAN (Call it a Night®)	Fucito et al., 2017	ALC	hcon		University	20,71 / 20,33
**12**	College Alc	Bersamin et al., 2007	ALC	all		University	18
**13**	D_ARIANNA ((Digital-Alcohol Risk Alertness Notifying Network for Adolescents and Young Adults))	Carrà et al., 2016	ALC	hcon		General population	20.6
**14**	DrAFT-CS (Drinking Assessment and Feedback Tool for College Students)	Wagener et al., 2012	ALC	hcon		University	20.28
		Weaver et al., 2014	ALC	con		University	n.r.
**15**	ECALC (expectancy challenge alcohol literacy curriculum)	Dunn et al., 2020	ALC	hcon		University	19.42
**16**	eCHUG (electronic version of Check-up to go)	Alfonso et al., 2012	ALC	hcon		University	18.77
		Braitman et al., 2022	ALC	hcon		University	19.9
		Doumas et al., 2011	ALC	hcon		University	19.7
		Hustad et al., 2010	ALC	all		University	18.1
		Thompson et al., 2018	ALC	all		University	17.8
		Tahaney & Palfay, 2017	ALC	hcon		University	18.99
		Walters et al., 2007	ALC	hcon		University	19.8
**17**	eentjeteveel	Fraeyman et al., 2012	ALC	all		University	19
**18**	eSBI (electronic screening and brief intervention)	Kypri et al., 2013	ALC	all	Maori students	University	20.2
		Kypri et al., 2014	ALC	con	Ethnicity other than Maori	University	20.3
		Kypri et al., 2008	ALC	hcon	Patient in University health care center	University	20.1
**19**	ETUCARE	Theurel et al., 2022	ALC	all		University	20.19
**20**	FIT (functional imagery training)	Shuai et al., 2022	ALC	hcon		General population	20.63
**21**	M-PASS (Michigan Prevention and Alcohol Safety for Students)	Bingham et al., 2011	ALC	con	International students	University	18-20
**22**	MyStudentBody:Alcohol	Chiauzzi et al., 2005	ALC	hcon		University	19.9
**23**	myPlaybook	Fearnow-Kenney et al., 2016	ALC	con	Athletes	University	18.43
		Zamboanga et al., 2019	ALC	con	Athletes	General population	18.8

**24**	PantherTRAC	Suffoletto et al., 2016	ALC	hcon		University	n.r.
**25**	PartyWise	Schwarz et al., 2022	ALC	all	Females using highly effective birthcontrol	General population	18
**26**	Ray's Night Out	Hides et al., 2018	ALC	con		General population	20.4
		Pocuca et al., 2016	ALC	hcon		University	20.67
**27**	THRIVE (Tertiary Health Research Intervention via Email)	Kypri et al., 2009	ALC	hcon		University	19.7
		Leeman et al., 2016	ALC	hcon		University	19.85
**60**	Unitcheck	Bewick et al., 2013	ALC	con		University	20.8
		Marley et al., 2016	ALC	hcon		University	19.33
**29**	WDYD (What do you drink?)	Voogt et al., 2014a	ALC	hcon		University	20.8
**30**	Y1-CAP (Year 1 College Alcohol Profile)	Shell et al., 2019	ALC	all		University	n.r.
**31**	No name	Bernstein et al., 2018	ALC	con	21st birthday approaching	University	21
**32**	No name	Bonar et al., 2022	ALC	hcon		General population	20.4
**33**	*No name*	Braitman et al., 2020	ALC	con		University	19.65
**34**	*No name*	Boyle et al., 2021	ALC	con		University	18.05
**35**	*No name*	Bryant et al., 2013	ALC	all		University	18.7
**36**	*No name*	Buckner et al., 2019	ALC	con	Intention to consume at Mardi Gras	University	20.08
**37**	*No name*	Butler et al., 2009	ALC	hcon		University	19,7/ 20,6/ 20,38
**38**	*No name*	Carey et al., 2020	ALC	hcon		University	18
**39**	*No name*	Collins et al., 2014	ALC	hcon		University	20.78
**40**	*No name*	Doumas et al., 2009	ALC	hcon		University	19.24
**41**	*No name*	Ellis et al., 2017	ALC	con		University	n.r.
**42**	*No name*	Gex et al., 2023	ALC	hcon		University	19.95
**43**	*No name*	Glowacki et al., 2020	ALC	all		University	19.4
**44**	*No name*	Hagger et al., 2012	ALC	all		University	20.11

**45**	*No name*	Hendershot et al., 2010	ALC	all	Northeastern Asian descent (Chinese, Korean or Japanese heritage)	University	20.2
**46**	*No name*	Kazemi et al., 2020 (study 1)	ALC	hcon		University	19.04
**47**	*No name*	Kazemi et al., 2020 (study 2)	ALC	con		University	19.86
**48**	*No name*	LaBrie et al., 2019	ALC	hcon		University	n.r.
**49**	*No name*	Larimer et al., 2023	ALC	hcon		University	20.13
**50**	*No name*	Leary et al., 2022	ALC	con		University	18.09
**51**	*No name*	Leavens et al., 2020	ALC	hcon		University	18.09
**52**	*No name*	Lewis et al., 2007	ALC	hcon		University	18.53
**53**	*No name*	Ma, 2022	ALC	all		University	19.06
**54**	*No name*	Mason et al., 2014	ALC	hcon		University	19.2
**55**	No name	McGeary et al., 2014	ALC	hcon	Males only	University	18.98
**56**	*No name*	Neighbors et al., 2009	ALC	con	Approaching 21st birthday	University	21
**57**	*No name*	Norman et al., 2018	ALC	all		University	18.76
**58**	*No name*	Palfai et al., 2011	ALC	hcon		University	18.6
**59**	*No name*	Palfai et al., 2014a	ALC	all		University	18.21
**60**	*No name*	Pilling et al., 2007	ALC	all		University	19.05
**61**	*No name*	Ridout & Cambell, 2014	ALC	hcon		University	19.05
**62**	*No name*	Riordan et al., 2023	ALC	all		University	18.06
**63**	*No name*	Savage et al., 2015	ALC	con		University	18.51
**64**	*No name*	Schuckit et al., 2016	ALC	hcon		University	18.00
**65**	*No name*	Smallman et al., 2023	ALC	con		University	18.97
**66**	*No name*	Spijkerman et al., 2010	ALC	hcon		General population	18.1
**67**	*No name*	Tello et al., 2018	ALC	all		University	19.84
**68**	*No name*	Thombs et al., 2007	ALC	all		University	n.r.
**69**	*No name*	Walters et al., 2009	ALC	hcon		University	19.8
**70**	*No name*	Whitt et al., 2019	ALC	con		University	21
**71**	*No name*	Yang & Nan, 2019	ALC	con		University	19.77

**72**	Mobile Coach Tobacco & Mobile Coach Tobacco +	Haug et al., 2017a	TOB	con		Vocational school	18.6
**73**	BASICS (Brief Alcohol Screening and Intervention for College Students - adaption for marijuana)	Lee et al., 2010	CAN	con		University	18.03
**74**	CAAT (Cannabis Approach Avoidance Training)	Karoly et al., 2019	CAN	con		General population	18.83
**75**	eCHUG (electronic version of Check-up to go)marijuana	Elliott et al., 2014	CAN	all	Psycholgy students	University	19.34
		Palfai et al., 2014b	CAN	con	In student health services	University	19.33-20.33
**76**	*No name*	Buckner et al., 2020	CAN	con		University	19.1
**77**	*No name*	Walukevich-Dienst et al., 2019	CAN	hcon		University	19.83
**78**	iHeLP	Braciszewski et al., 2018	substance of choice	hcon	No more than 2 years removed from foster care; moderate or sever risk of alc, smoking, substance involvement (ASSIST); not in treatment;	General population	18.91
**79**	d-SBI (Digital screening and brief intervention for alcohol misuse)	Sharma et al., 2023	CAN, 'illicit drugs'	hcon		University	20.5
**80**	U@Uni:LifeGuide	Cameron et al., 2015	ALC, TOB	all		University	18.73
**81**	CAAT (Cannabis Approach Avoidance Training)	Jacobus et al., 2018	ALC, CAN	con		General population	19
**82**	MiSARA (substance abuse research assistant)	Coughlin et al., 2021	ALC, CAN	hcon	Past month binge drinking OR cannabis use	General population	20.7
**83**	myPlaybook	Rulison et al., 2022 (study 1)	ALC, TOB, CAN, performance enhancing drugs/dietary supplements, prescription/over-the-counter drugs	all	Athletes	University	n.r.
		Rulison et al., 2022 (study 2)	ALC, CAN	all	Athletes	University	n.r.
		Rulison et al., 2022 (study 3)	ALC, CAN	all	Athletes	University	n.r.

**84**	Meine Zeit ohne - Die Challenge	Pietsch et al., 2023	ALC, TOB, eTOB, CAN	all		Vocational school	19.2
**85**	Project Fitness	Moore et al., 2012	ALC, TOB, CAN & various health behaviours	all		University	19.44

* Target substance; ALC = alcohol, TOB = tobacco (smoking), eTOB = vaping, CAN = cannabis (ncl. marijuana), ECT = ecstasy, MET = methamphetamines, HAL = hallucinogenes, NPD = new psychoactive drugs, OTC = over-the-counter drugs, PD = prescription drugs, DU = 'drug use' (various/unspecified)

** Consumption patterns: all = consumers & non-consumers, con = consumers (abstinent persons excluded), hcon = heavy consumers or mandate students

*** n.r. = not reported

Supplementary file S8_Mode of delivery, components and underlying theory of digital interventions

	Project / intervention	Publication	Theoretical bases of e-intervention*	Mode of delivery**	Components														Additional non-digital components
					Information/education (text, figures, videos)	Non-interactive video	Interactive video	Virtual reality 3D (hmd)	Avatar / interactive video	Chatroom / discussion with peers	Chatroom / discussion with experts	Personal message / feedback	Chatbot	Quiz	Contest	Games	Exercise/training/tasks	Other	
**A Adolsecents**																			
**1**	Alc-Check	Haug et al., 2013	PNF	web, tm	x						x								
**2**	Alcohol Alert / Alerta Alcohol	Jander et al., 2016	I-Change model	web												x			
		Martinez-Montilla et al., 2020	I-Change model	web												x			
		Vargas-Martinez et al., 2019	I-Change model	web												x			
**3**	Click City®: Alcohol	Gordon et al., 2017	Health cognition models; cognitive theories of Ajzen & Fishbein; Slovic's risk perception & decision; Fiilmore's relation of behavioral disinhibition and alc consumption;	web	x					x				x		x			
**4**	Climate Schools	Newton et al., 2022a	hp, skill development & social influence	web	x		x												Combined in CAP
		Vogl et al., 2009	hp, SCT	web	x		x												Teacher-dilvered classroom interactive activity
**5**	eCHUG (electronic version of Check-up to go)	Doumas et al., 2019	PNF, hp	web								x							
		Doumas et al., 2020a	PNF, hp	web								x							
		Doumas et al., 2020b	PNF, hp	web								x							
		Doumas et al., 2021	PNF, info, hp	web								x							Members of research team present to instruct & assist
**6**	HSD (Healthy School and Drugs)	Koning et al., 2011	SCT, theory of planned behaviour	web													x		1 arm: parent intervention non-digital (1 arm combined with student intervention)
**7**	kiR (keepin' it REAL)	Shin et al., 2018	SCT, narrative engagement theory	web		x													

**8**	MobileCoach Alcohol	Haug et al., 2017b	PNF, health action process approach: outcome expectations, self-efficacy, and planning processes	web, tm	x				x					
		Haug et al., 2020	MI, info, hp, SCT	app	x				x					
		Haug et al., 2023	PNF, MI, info, hp, SCT	web & chatbot (app)	x				x	x	x			
**9**	PALMSS (peer alcohol knowledge, low-risk drinking, media-influence, social drinking, self-efficacy)	Hongthon et al., 2016	info, hp	CD	x									
**10**	Smart Choices 4 Teens	Byrnes et al., 2019	info, hp	web			x							Off-line semi-structured discussions parents with teens on hypothetical scenarios
**11**	SPORT	Mathews et al., 2007	?	CD, web					x				Contract at end	
**12**	VR *FestLab* (virtual reality FestLab)	Guldager et al., 2022	Behaviour change wheel	app, smartphone, hmd				x						
**13**	WDYD (What do you drink?)	Voogt et al., 2013	PNF, info, theory of planned behaviour: goal setting, action planning? part of I-Change model: knowledge, social norms & self-efficacy as most changeable determinants	web					x					
**14**	*No name*	Ho et al., 2021	info	web	x					x				
**15**	*No name*	Knight et al., 2018	info	pc	x				x					In-person councelling after intervention
**16**	*No name*	Russell et al., 2017	Social marketing approach, narrative persuasion & counter-advertising	pc		x								
**17**	*No name*	Schinke et al., 2009b	Family interaction theory	CD or web		x								
**18**	C2Q-Teen (Craving to Quit-Teen)	Pbert et al., 2020	mindfulness training (based on operant conditioning), info	app	x	x						x		Ongoing personal support from school-nurse
**19**	QuitSTART smoking app	Pbert et al., 2020	PNF	app					x				Monitoring	Ongoing personal support from school-nurse
**20**	*No name*	Kong et al., 2017	hp, contingecy management - reinforcements	tm										Weekly in-person cognitive behavioural therapy part of intervention!

**21**	*No name*	Mason et al., 2015	PNF, MI, TTM	tm	x					x					
**22**	*No name*	Shi et al., 2013	TTM, hp	tm, app, smartphone						x					
**23**	Invite Only VR (virtual reality)	Weser et al., 2021	info, hp	hmd				x				x			
**24**	smokeSCREEN	Bteddini et al., 2023	info, hp, SCT	pc			x					x			
**25**	xkpts.com ((reader: “perquèpetes.com”, translation: “why joints?”)	Ariza et al., 2013	info, hp, Attitudes-Social-Influences-Self-Efficacy (ASE) model	CD, web	x	x									Various classroom activities
**26**	P4T (POP4Teens) vs JTT (JustThinkTwice)	Champion et al., 2018	PNF, info, hp, Attitudes-Social-Influences-self-Efficacy (ASE) model	web	x		x								Teacher-led activities in classroom(discussions, work-sheets, role-play)
**27**	Elm City Story (PlayForward)	Marsch et al., 2021	info, hp	web	x						x		x		
**28**	REAL media	Montanaro et al., 2015	info, hp, SCT, message framing prospect theory	app			x					x			
**29**	*No name*	Greene et al., 2020	Theory of active involvement (media literacy)	web	x	x					x		x		
**30**	E-MOVO (Dutch for Electronic Monitoring and Health Promotion)	Fang & Schinke, 2013	Family interaction theory	web	x	x			x				x		
**31**	Health4Life	Crutzen et al., 2008	info	web	x					x					
**32**	Climate Schools	Champion et al., 2023	PNF, MI, info, SCT, self-determination theory	web, app	x										Optional: teacher-delivered activities (eg, worksheets, group discussions, and homework tasks)
		Champion et al., 2016	info, hp, Attitudes-Social-Influences-self-Efficacy (ASE) model	web	x		x								Teacher-led activities in classroom(discussions, work-sheets, role-play)
**33**	*No name*	Newton et al., 2022b	info, hp	web	x		x								Teacher-driven classroom activity
**34**	*No name*	Gryczynski et al., 2021	PNF, MI, info	pc	x		x								
**35**	Healthy School and Drugs	Knight et al., 2019	MI, info	pc	x										Clinician brief advice
**36**	¿Qu é pasa si te pasas? (What happens if you go to far?)	Malmberg et al., 2014	info, hp, SCT, theory of reasoned action	web	x	x			x	x			x	Contract at end	2nd arm: integral condition
**37**	ready4life	Fuentes et al., 2023	info, hp	app on pc	x	x					x	x	x		Teachers trained to manage intervention & emotions that might occur

**38**	SmartCoach	Haug et al., 2022	PNF, MI, SCT, general life-skills training, cognitive &-behavioural training	app, chatbot (smartphone)				**X**	Virtual coach	**X**	**X**				
**39**	RealTeen	Paz Castro et al., 2022	PNF, SCT, life-skills training	tm, app, chatbot (smartphone)	**X**			**X**		**X**	**X**			Several interactive features	
		Schwinn et al., 2019	hp, SCT	web	**X**	**X**				**X**			**X**	Non-drug-realted content	
**40**	Vamos	Schwinn et al., 2010	info, hp	web or CD	**X**	**X**				**X**					
**41**	*No name (family interaction)*	Schwinn et al., 2021	MI, hp, SCT	smartphone app		**X**		**X**					**X**		
		Schinke et al., 2009a	Family interaction theory, PNF, hp	pc or CD	**X**	**X**							**X**		
**42**	*No name*	Schinke et al., 2011	Family interaction theory; incorporated elements of social learning, attachment, and deviant behavior proneness theories , SCT	pc or CD	**X**	**X**							**X**		
**43**	E-health4Uth	Schwinn et al., 2015	Social competence and skill-building strategy, minority stress theory, hp	web or CD	**X**	**X**						**X**	**X**	Role play, writing activities	
**44**	Your Decision Counts TM	Bannink et al., 2014	PNF, reinforcement	web	**X**			**X**							2nd arm: & consultations
**45**	WISEteens	Evers et al., 2012	PNF, TTNM	web	**X**	**X**		**X**							School& family guides
**46**	Girls and Stress	Arnaud et al., 2016	PNF, MI, info	web	**X**	**X**		**X**							
**47**	*No name*	Schinke et al., 2005	?	CD			**X**								
**48**	Pure Rush	Williams et al., 2005	PNF, info, hp	CD			**X**								
**49**	Climate Schools Psychostimulant & Cannabis Modlue	Stapinski et al., 2018	info, motivational messages	web			**X**					**X**			Accompanying information booklet to be read beforehand
**50**	Refuse to Use	Vogl et al., 2014	info, hp, SCT	pc	**X**										Program includes classroom interactive activity delivered by teacher
**51**	Climate Schools: Ecstasy and Emerging Drugs Module	Duncan et al., 2020	info	CD			**X**								
**26**	Climate Schools: Exstasy and Emerging Drugs Module	Champion et al., 2018	PNF, info, hp, Attitudes-Social-Influences-self-Efficacy (ASE) model	web	**X**		**X**								Teacher-led activities in classroom(discussions, work-sheets, role-play)
**B Emerging adults**															

**1**	Alcohol 101 Plus™	Carey et al., 2011 (study 2)	info, hp	CD		x	x			x	
**2**	Alcohol 101	Barnett et al., 2007	PNF	CD		x	x				
		Braitman et al., 2016	PNF, info, hp	web, mail (booster)		x	x				
		Carey et al., 2009	info	CD		x	x			x	
**3**	Alcohol Edu® for Sanctions	Carey et al., 2011 (study 1)	info	CD	x						
**4**	Alcohol Edu® for College	Croom et al., 2009	info	web	x						
		Hustad et al., 2010 (A)	PNF, info, hp	web	x						
		Paschall et al., 2014	PNF, info, hp, SCT, theory of planned behaviour	web	x						
**5**	Alcohol-Wise	Croom et al., 2015 (study 1)	PNF, info	web	x				x		
		Croom et al., 2015 (study 2)	PNF	web	x				x		
		Gilbertson et al., 2017	PNF, info, cognitive behavioural strategies	web	x				x		
		Strohman et al., 2016	PNF, info, hp	web	x				x		
**6**	alcooquizz.ch (1)	Bertholet et al., 2015	PNF, info	mail, pc	x			x			
		Bertholet et al., 2016	PNF, info	mail, pc	x			x			
		Bertholet et al., 2018	PNF, info	mail, pc	x			x			
**7**	BASICS (Brief Alcohol Screening and Intervention for College Students)	Lee et al., 2014	PNF, MI, info	web	x			x			
		Linowski et al., 2016	PNF (MI face-to-face)	web	x			x			Primary intervention: BASICS offline (intervention & control group)
		Murphy et al., 2010, (study 1)	PNF, MI, hp	web	x			x			
		Murphy et al., 2010 (study 2)	PNF, MI, hp	web	x			x			
		Patrick et al., 2014	PNF, hp, 'encouragement' / advice	web	x			x			
		Patrick et al., 2021	PNF, self-monitoring support (stage 1), MI (stage 2)	mail, web	x			x			
		Saitz et al., 2007	PNF, MI , self-change approaches	web	x			x			
**8**	BASICS (Brief Alcohol Screening and Intervention for College	LaBrie et al., 2013	PNF, MI	web	x			x			
		Larimer et al., 2021	PNF, info, hp	web	x			x			

	Students) - PNF component	Moreira et al., 2012	PNF	mail	x					x				
		Neighbors et al., 2004	PNF	web	x					x				
		Neighbors et al., 2006	PNF	web	x					x				
		Neighbors et al., 2010	PNF	web	x					x				
**9**	Campus GANDR (Gamified Alcohol Norm Discovery & Readjustment)	Boyle et al., 2017	PNF, info	fb app						x		x		
**10**	CDCU (College Drinker's Check-up)	Carey et al., 2017	PNF, TTM	web									x	
		Hester et al., 2012 (study 1)	PNF, MI	web									x	
		Hester et al., 2012 (study 2)	PNF, MI	web									x	
**11**	CIAN (Call it a Night®)	Fucito et al., 2017	PNF, info, hp	web	x								x	
**12**	College Alc	Bersamin et al., 2007	PNF, info, hp	web	x			x	x	x	x			
**13**	D_ARIANNA ((Digital-Alcohol Risk Alertness Notifying Network for Adolescents and Young Adults))	Carrà et al., 2016	PNF, info, hp	app						x				
**14**	DrAFT-CS (Drinking Assessment and Feedback Tool for College Students)	Wagener et al., 2012	PNF, MI	web		x	x							
		Weaver et al., 2014	PNF, hp	web		x	x							
**15**	ECALC (expectancy challenge alcohol literacy curriculum)	Dunn et al., 2020 (B) 2019?	info	web	x									Clinical assistance during intervention
**16**	eCHUG (electronic version of Check-up to go)	Alfonso et al., 2012	PNF, MI	pc	x					x				
		Braitman et al., 2022	PNF, hp	mail	x					x				
		Doumas et al., 2011	PNF, MI	web						x				
		Hustad et al., 2010 (B)	PNF, info, hp	web	x					x				
		Thompson et al., 2018	PNF	web	x					x				
		Tahaney & Palfay, 2017	PNF, info, hp	web, tm	x					x				
		Walters et al., 2007	PNF	web	x					x				
**17**	eentjeteveel	Fraeyman et al., 2012	PNF, info, hp	web	x					x				
**18**		Kypri et al., 2013	PNF	web	x					x				

	eSBI (electronic screening and brief intervention)	Kypri et al., 2014	PNF	web	x						x			
		Kypri et al., 2008	PNF	web	x						x			
**19**	ETUCARE	Theurel et al., 2022	info, encouragement, mindfulness, cognitive behavioural therapy	web	x	x						x	x	
**20**	FIT (functional imagery training)	Shuai et al., 2022	FIT (functional imagery Training) - negative effect-focussed	ppt video	x						x			
**21**	M-PASS (Michigan Prevention and Alcohol Safety for Students)	Bingham et al., 2011	MI, TTM, hp, Health Belief Model, Theory of Planned Behaviour, Precaution Adoption Process Model	web			x	x			x			
**22**	MyStudentBody:Alcohol	Chiauzzi et al., 2005	PNF, MI, info, hp,	web	x		x	x			x			
**23**	myPlaybook	Fearnow-Kenney et al., 2016	PNF, Health Belief Model, Theory of Planned Behaviour, Theory of Reasoned Action	web	x						x			
		Zamboanga et al., 2019	PNF, MI, info, hp,	web	x						x			
**24**	PantherTRAC	Suffoletto et al., 2016	PNF	tm							x			Adjunct to classroom sessions
**25**	PartyWise	Schwarz et al., 2022	MI, I-change model	tm, pc	x						x			Telephone screening as part of intervention
**26**	Ray's Night Out	Hides et al., 2018	MI, info, Information-Motivation-Behavioral skills model	app			x	x						
		Pocuca et al., 2016	hp	app			x	x						
**27**	THRIVE (Tertiary Health Research Intervention via Email)	Kypri et al., 2009	PNF, MI	web							x			
		Leeman et al., 2016	PNF, MI, hp	web							x			
**60**	Unitcheck	Bewick et al., 2013	PNF, MI, hp	web							x			
		Marley et al., 2016	PNF	web							x			
**29**	WDYD (What do you drink?)	Voogt et al., 2014a	PNF, MI, info, hp, I-change model	web							x			
**30**	Y1-CAP (Year 1 College Alcohol Profile)	Shell et al., 2019	PNF, reinforcement (for abstainers)	web							x			
**31**	No name	Bernstein et al., 2018	PNF	tm							x			
**32**	No name	Bonar et al., 2022	group MI, cognitive behavioural therapy	fb (secret groups)	x				x	x				
**33**	*No name*	Braitman et al., 2020	PNF, hp	web, mail (booster)							booster			

**34**	*No name*	Boyle et al., 2021	PNF	web, social media						**X**	
**35**	*No name*	Bryant et al., 2013	PNF	mail					**X**		
**36**	*No name*	Buckner et al., 2019	PNF	web					**X**		
**37**	*No name*	Butler et al., 2009	PNF, (MI in face-to-face intervention)	pc	**X**				**X**		
**38**	*No name*	Carey et al., 2020	PNF	tm					**X**		
**39**	*No name*	Collins et al., 2014	PNF, decisional balance feedback (DBF)	web					**X**		
**40**	*No name*	Doumas et al., 2009	PNF	web	**X**				**X**		
**41**	*No name*	Ellis et al., 2017	PNF, MI	pc	**X**				**X**		
**42**	*No name*	Gex et al., 2023	PNF, MI, 'substance-free activity'	tm					**X**		
**43**	*No name*	Glowacki et al., 2020	PNF, regulatory focus fit	tm					**X**		
**44**	*No name*	Hagger et al., 2012	Theory of Planned Behaviour; motivation & inplementation / action-phase model: mental simulation (present/absent) x implementation intention (present/absent)	web							**X**
**45**	*No name*	Hendershot et al., 2010	individualized genetic feedback !!!	web					Genetic feedback		
**46**	*No name*	Kazemi et al., 2020 (study 1)	PNF, MI, info, TTM, hp, reinforcement	app					**X**		
**47**	*No name*	Kazemi et al., 2020 (study 2)	PNF, MI, info, TTM, hp, reinforcement	app					**X**		
**48**	*No name*	LaBrie et al., 2019	PNF	web						**X**	
**49**	*No name*	Larimer et al., 2023	PNF, info, hp	web	**X**				**X**		
**50**	*No name*	Leary et al., 2022	Deviance regulation theory	web					**X**		
**51**	*No name*	Leavens et al., 2020	PNF	web, mail	**X**				**X**		
**52**	*No name*	Lewis et al., 2007	PNF	pc	**X**				**X**		
**53**	*No name*	Ma, 2022	Immersive storytelling	pc, hmd (i2)		i1	i2	i1			
**54**	*No name*	Mason et al., 2014	PNF, MI, TTM	tm							
**55**	No name	McGeary et al., 2014	Attention modification	web							**X**

**56**	*No name*	Neighbors et al., 2009	PNF	web	**X**		**X**					
**57**	*No name*	Norman et al., 2018	hp, intervention 1) self-affirmation manipulation, intervention 2 Theory of Planned Behaviour	web			**X**				Self-affirmation; implementation intention	
**58**	*No name*	Palfai et al., 2011	PNF	web	**X**		**X**					Part of assessment via paper-pencil
**59**	*No name*	Palfai et al., 2014a	PNF	web	**X**		**X**					
**60**	*No name*	Pilling et al., 2007	PNF, schema-based approaches	web	**X**		**X**					
**61**	*No name*	Ridout & Cambell, 2014	PNF	web	**X**		**X**					
**62**	*No name*	Riordan et al., 2023	PNF (wbi),, hp, (social costs of excessive drinking messages)	web / tm (EMI)	**X**		**X**					
**63**	*No name*	Savage et al., 2015	PNF, info, hp	web	**X**			**X**				
**64**	*No name*	Schuckit et al., 2016	PNF, MI	web	**X**			**X**				
**65**	*No name*	Smallman et al., 2023	hp, functional theory of counterfactual thinking	web						Counterfactuals		
**66**	*No name*	Spijkerman et al., 2010	PNF	web	**X**		**X**					
**67**	*No name*	Tello et al., 2018	Evaluative conditioning	web						Evaluative conditioning		
**68**	*No name*	Thombs et al., 2007	PNF	web	**X**	**X**	**X**					
**69**	*No name*	Walters et al., 2009	PNF, MI	web	**X**		**X**					
**70**	*No name*	Whitt et al., 2019	PNF	web, tm			**X**					
**71**	*No name*	Yang & Nan, 2019	PNF	web	**X**		**X**					
**72**	Mobile Coach Tobacco & Mobile Coach Tobacco +	Haug et al., 2017a	PNF (alc, SCT, HAPA (health action process aproach)	web, tm	**X**		**X**	**X**	**X**			
**73**	BASICS (Brief Alcohol Screening and Intervention for College Students - adaption for marijuana)	Lee et al., 2010	PNF, MI, hp	web	**X**							
**74**	CAAT (Cannabis Approach Avoidance Training)	Karoly et al., 2019	Cognitive bias modification	web						**X**		
**75**	eCHUG (electronic version of Check-up to go)marijuana	Elliott et al., 2014	PNF, info, hp	web			**X**					With clinical assistance
		Palfai et al., 2014b	PNF, info, hp	web			**X**					

**76**	*No name*	Sharma et al., 2023	PNF, 'encouragement' / advice / provision of personal strategies	web or app	x	x		
**77**	*No name*	Buckner et al., 2020	PNF, PFI-NAC: encouragement, including strategies to manage negative effects	web	x	x		
**78**	iHeLP	Walukevich-Dienst et al., 2019	PNF, info	web	x	x		
**79**	d-SBI (Digital screening and brief intervention for alcohol misuse)	Braciszewski et al., 2018	PNF, MI, info, TTM, hp, FRAMES approach	tm, mp, pc	x	x		
**80**	U@Uni:LifeGuide	Cameron et al., 2015	self-affirmation manipulation, theory of planned behaviour and implementation intention task	web				
**81**	CAAT (Cannabis Approach Avoidance Training)	Jacobus et al., 2018	Cognitive bias modification	pc				x
**82**	MiSARA (substance abuse research assistant)	Coughlin et al., 2021	PNF, MI, reciprocity strategy	app		x		x
**83**	myPlaybook	Rulison et al., 2022 (study 1)	PNF, theory of reasoned action & health belief model	web	x	x		
		Rulison et al., 2022 (study 2)	PNF, theory of reasoned action & health belief model	web	x	x		
		Rulison et al., 2022 (study 3)	PNF, hp, theory of reasoned action & health belief model	web	x	x		
**84**	Meine Zeit ohne - Die Challenge	Pietsch et al., 2023	MI, voluntary abstinence paradigm	app	x		x	
**85**	Project Fitness	Moore et al., 2012	Behavior Image Model (BIM)	pc	x	x		

* PNF = personal (normative) feedback, MI = motivational interviewing, info = information / education / health literacy, TTM = transtheoretical model/readiness to change, hp = harm prevention / personal behavioural strategies, SCT = social cognitive theory

**web = web-based, pc =(personal) computer, CD = CD rom, app = application (phone or web), fb = facebook, hmd = head mounted display (VR 3D), tm = text message,

Supplementary file S9_Evidence assessment

	Project / intervention	Publication	Study design*	Latest follow-up (in months)	Outcome measures												Intervention components		
					Consumption	Binge/heavy drinking episodes	Substance-related problems	Intention to consume	Motivation / readiness to change	Perception of peer consumption	Norms & attitudes	Consumption effects expectancies	Knowledge	Protective behavioural strategies	Self-efficacy	Other	Single component intervention	Multi Evaluation combined	Multi Evaluation separate
**A Adolescents**																			
**1**	Alc-Check	Haug et al., 2013	p-p	3	X	X	X		X									X	
**2**	Alcohol Alert / Alerta Alcohol	Jander et al., 2016	RCT	4	X	X			X									X	
		Martinez-Montilla et al., 2020	RCT	4	X	X												X	
		Vargas-Martinez et al., 2019	RCT	4	X	X										health-related quality of life		X	
**3**	Click City®: Alcohol	Gordon et al., 2017	RCT	0.25				X		X						risk behaviour: willingness to drive after drinking		X	
**4**	Climate Schools	Newton et al., 2022a	RCT	84	X	X	X											X	
		Vogl et al., 2009	RCT	12	X		X					X	X					X	
**5**	eCHUG (electronic version of Check-up to go)	Doumas et al., 2019	RCT	6			X				X							X	
		Doumas et al., 2020a	RCT	1	X	X				X		X		X				X	
		Doumas et al., 2020b	p-p	1	X	X												X	
		Doumas et al., 2021	RCT	6	X	X											X		
**6**	HSD (Healthy School and Drugs)	Koning et al., 2011	RCT	34	X	X												X	
**7**	kiR (keepin' it REAL)	Shin et al., 2018	RCT	1	X										X			X	
**8**	MobileCoach Alcohol	Haug et al., 2017b	RCT	6	X	X				X								X	

		Haug et al., 2020	RCT	0	x	x									smoking		x
		Haug et al., 2023	RCT	0	x	x								x			x
**9**	PALMSS (peer alcohol knowledge, low-risk drinking, media-influence, social drinking, self-efficacy)	Hongthon et al., 2016	RCT	6								x			advertising exposure, family drinking		x
**10**	Smart Choices 4 Teens	Byrnes et al., 2019	RCT	12	x	x			x	x					family communication regarding teen alc use & parent drinking		x
**11**	SPORT	Mathews et al., 2007	RCT	0			x								images of those who use alcohol, beliefs about linkage between alcohol & other health promoting behaviours		x
**12**	VR *FestLab* (virtual reality FestLab)	Guldager et al., 2022	RCT	1.5		x					x	x		x	Susceptibility to peer pressure, sensation-seeking, communication skills, social support willingness		x
**13**	WDYD (What do you drink?)	Voogt et al., 2013	RCT	6	x	x											x
**14**	*No name*	Ho et al., 2021	RCT	3	x					x		x			Behavioural control	x	
**15**	*No name*	Knight et al., 2018	p-p	12	x	x											x
**16**	*No name*	Russell et al., 2017	RCT	0			x			x							x
**17**	*No name*	Schinke et al., 2009b	RCT	2	x		x		x				x		Parental monitoring, rules & communication		x
**18**	C2Q-Teen (Craving to Quit-Teen)	Pbert et al., 2020	RCT	6	x												x
**19**	QuitSTART smoking app	Pbert et al., 2020	RCT	6	x												x
**20**	*No name*	Kong et al., 2017	p-p	1	x												x
**21**	*No name*	Mason et al., 2015	RCT	6	x		x	x	x						Risk behaviour, peer social support, family smoking context		x

**22**	*No name*	Shi et al., 2013	RCT	0	x				x		x	x			Stage of cessation	x
**23**	Invite Only VR (virtual reality)	Weser et al., 2021	RCT	6	x						x	x		x		x
**24**	smokeSCREEN	Bteddini et al., 2023	sequential mixed methods design	4.25	x		x	x			x	x				x
**25**	xkpts.com ((reader: “perquèpetes.com”, translation: “why joints?”)	Ariza et al., 2013	quasi-experimental	15	x											x
**26**	P4T (POP4Teens) vs JTT (JustThinkTwice)	Marsch et al., 2021	RCT	6				x			x	x			Risk perceeption, skill acquisition	x
**27**	Elm City Story (PlayForward)	Montanaro et al., 2015	RCT	6								x				x
**28**	REAL media	Greene et al., 2020	RCT	3							x			x		x
**29**	*No name*	Fang & Schinke, 2013	RCT	24	x								x	x	Mother-daughter closeness, communication, maternal monitoring & family rules against substance use	x
**30**	E-MOVO (Dutch for Electronic Monitoring and Health Promotion)	Crutzen et al., 2008	p-p	2				x								x
**31**	Health4Life	Champion et al., 2023	RCT	24	x											x
**32**	Climate Schools	Champion et al., 2016	RCT	0	x	x		x				x				x
		Newton et al., 2022b	RCT	12	x	x	x					x				x
**33**	*No name*	Gryczynski et al., 2021	RCT	6	x	x	x								Unprotected sex & sex while intoxicated; health-related qualit of life, activity-based costs	x
**34**	*No name*	Knight et al., 2019	RCT	12	x	x	x			x						x
**35**	Healthy School and Drugs	Malmberg et al., 2014	RCT	32	x											x
**36**	¿Qu é pasa si te pasas? (What happens if you go to far?)	Fuentes et al., 2023	p-p	0									x		Risk perception	x
**37**	ready4life	Haug et al., 2022	RCT	6	x	x								x	Internet use & self-perceived stress	x

**38**	SmartCoach	Paz Castro et al., 2022	RCT	18	x	x								Well-being, Social skills		x
**39**	RealTeen	Schwinn et al., 2019	RCT	36	x				x			x	x	Media literacy, anxious & depressive mood, body image, perceived stress, self-Esteem		x
		Schwinn et al., 2010	RCT	6	x				x			x	x	Social skills, stress management, body esteem, self esteem		x
**40**	Vamos	Schwinn et al., 2021	RCT	36	x				x			x	x	Literacy, mood		
**41**	*No name (family interaction)*	Schinke et al., 2009a	RCT	24	x		x		x			x	x	Self-perception, depression, family communication, closeness, rules, monitoring		x
		Schinke et al., 2011	RCT	0	x		x		x			x				x
**42**	*No name*	Schwinn et al., 2015	RCT	3	x				x			x		Perceived stress		x
**43**	E-health4Uth	Bannink et al., 2014	RCT	4	x	x								Well-being, health-related quality of life		x
**44**	Your Decision Counts TM	Evers et al., 2012	RCT	14	x			x								x
**45**	WISEteens	Arnaud et al., 2016	RCT	3	x	x									x	
**46**	Girls and Stress	Schinke et al., 2005	RCT	0.5			x							likely response to stress, capacity to handle stress, awareness of unhealthy ways to reduce stress, role of cigarettes, alcohol or drugs as responses to stress, satisfaction with current appearance & weight, concern about what friends think of them		x
**47**	*No name*	Williams et al., 2005	RCT	0	x	x			x	x	x					x
**48**	Pure Rush	Stapinski et al., 2018	RCT	0			x				x					x

**49**	Climate Schools Psychostimulant & Cannabis Modlue	Vogl et al., 2014	RCT	10	**x**			**x**			**x**		**x**					**x**
**50**	Refuse to Use	Duncan et al., 2020	p-p	0				**x**			**x**			**x**	**x**			**x**
**51**	Climate Schools: Ecstasy and Emerging Drugs Module	Champion et al., 2018	RCT	24	**x**			**x**					**x**					**x**
**B Emerging adults**																		
**1**	Alcohol 101 Plus™	Carey et al., 2011 (study 2)	RCT	12	**x**	**x**	**x**											**x**
**2**	Alcohol 101	Barnett et al., 2007	RCT	12	**x**	**x**	**x**		**x**	**x**				**x**		Help-seeking & Recidivism		**x**
		Braitman et al., 2016	RCT	1	**x**	**x**	**x**							**x**				**x**
		Carey et al., 2009	RCT	12	**x**	**x**	**x**		**x**									**x**
**3**	Alcohol Edu® for Sanctions	Carey et al., 2011 (study 1)	RCT	12	**x**	**x**	**x**											**x**
**4**	Alcohol Edu® for College	Croom et al., 2009	RCT	1.5	**x**		**x**						**x**	**x**				**x**
		Hustad et al., 2010 (A)	RCT	1	**x**	**x**	**x**											**x**
		Paschall et al., 2014	RCT	5	**x**						**x**	**x**		**x**				**x**
**5**	Alcohol-Wise	Croom et al., 2015 (study 1)	RCT	1.6	**x**								**x**			Forms of risk behavior		**x**
		Croom et al., 2015 (study 2)	RCT	1.6	**x**								**x**			Forms of risk behavior		**x**
		Gilbertson et al., 2017	p-p	6	**x**	**x**	**x**					**x**				Adaption to college		**x**
		Strohman et al., 2016	RCT	1	**x**	**x**				**x**		**x**						**x**
**6**	alcooquizz.ch (1)	Bertholet et al., 2015	RCT	6	**x**	**x**	**x**											**x**
		Bertholet et al., 2016	RCT	6		**x**					**x**							**x**
		Bertholet et al., 2018	RCT	47	x	**x**												**x**
**7**	BASICS (Brief Alcohol Screening and Intervention for College Students)	Lee et al., 2014	RCT	0.25	**x**		**x**	**x**		**x**	**x**	**x**						**x**
		Linowski et al., 2016	RCT	12	**x**	**x**	**x**										Booster	

		Murphy et al., 2010, (study 1)	RCT	1	x				x					Subjective change in drinking & normative & self-ideal discrepancies		x	
		Murphy et al., 2010 (study 2)	RCT	1	x				x					Subjective change in drinking & normative & self-ideal discrepancies		x	
		Patrick et al., 2014	RCT	0	x		x	x		x	x		x	Sexual behavior		x	
		Patrick et al., 2021	RCT	0	x	x	x							Utilization of health services		Intervention	Bridging strategies
		Saitz et al., 2007	RCT	1	x	x	x		x					Help-seeking & family history & age of consumption onset		x	
**8**	BASICS (Brief Alcohol Screening and Intervention for College Students) - PNF component	LaBrie et al., 2013	RCT	12	x		x			x					x		
		Larimer et al., 2021	RCT	12	x		x								x		
		Moreira et al., 2012	RCT	12	x	x	x			x		x			x		
		Neighbors et al., 2004	RCT	6	x	x	x			x		x			x		
		Neighbors et al., 2006	RCT	2	x	x	x			x					x		
		Neighbors et al., 2010	RCT	24	x	x	x			x					x		
**9**	Campus GANDR (Gamified Alcohol Norm Discovery & Readjustment)	Boyle et al., 2017	RCT	0.5	x	x					x					x	
**10**	CDCU (College Drinker's Check-up)	Carey et al., 2017	RCT	6	x	x	x									x	
		Hester et al., 2012 (study 1)	RCT	12	x	x	x							Family history of alc. Problems		x	
		Hester et al., 2012 (study 2)	RCT	1	x	x	x							Family histroy of alc. Problems		x	
**11**	CIAN (Call it a Night®)	Fucito et al., 2017	RCT	3	x		x						x	Sleep characteristics		x	
**12**	College Alc	Bersamin et al., 2007	RCT	3	x	x	x										
**13**	D_ARIANNA ((Digital-Alcohol Risk Alertness Notifying Network for	Carrà et al., 2016	p-p	0.5		x	x	x								x	

	Adolescents and Young Adults))																
14	DrAFT-CS (Drinking Assessment and Feedback Tool for College Students)	Wagener et al., 2012	RCT	2.5	X		X										X	
		Weaver et al., 2014	RCT	1	X	X										X		Moderation skills
15	ECALC (expectancy challenge alcohol literacy curriculum)	Dunn et al., 2020 (B) 2019?	Outcome comparison study; random assignment	1	X		X		X			X			Motivational Interviewing treatment integrity		X	
16	eCHUG (electronic version of Check-up to go)	Alfonso et al., 2012	RCT	3	X		X								Motivational interviewing treatment integrity		X	
		Braitman et al., 2022	RCT	3	X	X	X			X			X		Personal leasure time activities & goals		X	
		Doumas et al., 2011	RCT	8	X	X	X			X							X	
		Hustad et al., 2010 (B)	RCT	1	X	X	X										X	
		Thompson et al., 2018	RCT	5	X		X			X							X	
		Tahaney & Palfay, 2017	RCT	1	X		X										X	
		Walters et al., 2007	RCT	4	X		X										X	
17	eentjeteveel	Fraeyman et al., 2012	qual	0	X	X		X	X								X	
18	eSBI (electronic screening and brief intervention)	Kypri et al., 2013	RCT	5	X	X	X										X	
		Kypri et al., 2014	RCT	5	X	X	X										X	
		Kypri et al., 2008	RCT	12	X	X	X			X							X	
19	ETUCARE	Theurel et al., 2022	Non-radomized controlled trial	1	X										Mental health problems		X	
20	FIT (functional imagery training)	Shuai et al., 2022	RCT	0.5		X								X	Drinking motifs, anxiety, depression	X		
21	M-PASS (Michigan Prevention and Alcohol Safety for Students)	Bingham et al., 2011	Paired intervention-control design	3	X	X	X		X		X		X		Drinking motifs & risk behavior (drink driving & driving with drink-driver)		X	
22	MyStudentBody:Alcohol	Chiauzzi et al., 2005	RCT	3	X		X		X								X	

**23**	myPlaybook	Fearnow-Kenney et al., 2016	RCT	0						x		x		x				x
		Zamboanga et al., 2019	RCT	4	x	x												x
**24**	PantherTRAC	Suffoletto et al., 2016	p-p	0	x	x		x	x								x	
**25**	PartyWise	Schwarz et al., 2022	RCT	9		x	x							x				x
**26**	Ray's Night Out	Hides et al., 2018	RCT	6		x	x	x						x				x
		Pocuca et al., 2016	Mixed methods	0					x									x
**27**	THRIVE (Tertiary Health Research Intervention via Email)	Kypri et al., 2009	RCT	6	x		x									Help-seeking behavior & subjective treatment effects		x
		Leeman et al., 2016	RCT	6	x	x	x	x						x				x
**60**	Unitcheck	Bewick et al., 2013	RCT	8.5	x	x												x
		Marley et al., 2016	qual	0				(x)		(x)	(x)	(x)	(x)					x
**29**	WDYD (What do you drink?)	Voogt et al., 2014a	RCT	6	x	x									x			x
**30**	Y1-CAP (Year 1 College Alcohol Profile)	Shell et al., 2019	Completers vs non-completers	24	x		x			x						Retention (subsequent semester enrollment) & family history & spendings on alc.		x
**31**	No name	Bernstein et al., 2018	RCT	0	x		x				x			x				x
**32**	No name	Bonar et al., 2022	RCT	12		x		x								Risk behaviour: driving after drinking & cannabis consumption; general drug use index	x	
**33**	*No name*	Braitman et al., 2020	RCT	9	x	x				x				x				x
**34**	*No name*	Boyle et al., 2021	RCT	1	x	x				x						Social media-based alc. Exposure & frequency of posting alc.-related content		x
**35**	*No name*	Bryant et al., 2013	RCT	1.5	x	x				x							x	
**36**	*No name*	Buckner et al., 2019	RCT	1	x			x		x							x	
**37**	*No name*	Butler et al., 2009	RCT	1	x	x	x			x								x

**38**	*No name*	Carey et al., 2020	RCT	6	x	x	x			x	x				x		
**39**	*No name*	Collins et al., 2014	RCT	12	x	x	x			x				Decisional balance	x		
**40**	*No name*	Doumas et al., 2009	RCT	1	x	x	x			x					x		
**41**	*No name*	Ellis et al., 2017	RCT	0				x	x					Narrator empathy		x	Empathic narrator
**42**	*No name*	Gex et al., 2023	RCT	3	x	x	x									x	
**43**	*No name*	Glowacki et al., 2020	RCT	0	x	x								Regulatory focus	x		
**44**	*No name*	Hagger et al., 2012	RCT	1	x	x		x			x		x	Perceived behavioral control & motivation to keep consumption in safe limits		x	
**45**	*No name*	Hendershot et al., 2010	RCT	1	x			x	x			x		Risk perception, fear arousal		x	
**46**	*No name*	Kazemi et al., 2020 (study 1)	RCT	1.5	x	x	x		x				x			x	
**47**	*No name*	Kazemi et al., 2020 (study 2)	RCT	1.5	x	x	x		x				x			x	
**48**	*No name*	LaBrie et al., 2019	RCT	0.75	x	x				x				Copresence (social perception while thinking of others)			x
**49**	*No name*	Larimer et al., 2023	RCT	12	x	x	x			x						x	
**50**	*No name*	Leary et al., 2022	RCT	1.25	x		x				x		x		x		
**51**	*No name*	Leavens et al., 2020	RCT	1	x		x				x		x			x	
**52**	*No name*	Lewis et al., 2007	RCT	5	x					x					x		
**53**	*No name*	Ma, 2022	RCT	0							x			Risk behavior: intention to drive; perceived similarity to the protagonist	x		

**54**	*No name*	Mason et al., 2014	RCT	1	x	x			x			x		Difficulty stopping & psychiatric symptoms & social network satisfaction	x		
**55**	No name	McGeary et al., 2014	RCT	0	x	x									x		
**56**	*No name*	Neighbors et al., 2009	RCT	0.25	x	x				x			x			x	
**57**	*No name*	Norman et al., 2018	RCT	6	x	x								Conceptions about binge drinking			x
**58**	*No name*	Palfai et al., 2011	RCT	1	x	x	x							Various health behaviours		x	
**59**	*No name*	Palfai et al., 2014a	RCT	5	x	x	x							Various health behaviours	x		
**60**	*No name*	Pilling et al., 2007	RCT	0				x			x						x
**61**	*No name*	Ridout & Cambell, 2014	RCT	3	x					x	x				x		
**62**	*No name*	Riordan et al., 2023	RCT	8	x		x									x	
**63**	*No name*	Savage et al., 2015	RCT	5	x									AUD symptoms		x	
**64**	*No name*	Schuckit et al., 2016	RCT	12	x									Level of response & self-rated effects of alcohol & genetics of alcoholism		x	
**65**	*No name*	Smallman et al., 2023	RCT	1	x	x	x						x		x		
**66**	*No name*	Spijkerman et al., 2010	RCT	3	x											x	
**67**	*No name*	Tello et al., 2018	RCT	0	x	x						x			x		
**68**	*No name*	Thombs et al., 2007	RCT	?	x		x									x	
**69**	*No name*	Walters et al., 2009	RCT	6	x		x		x	x			x				x
**70**	*No name*	Whitt et al., 2019	RCT	0	x		x	x						Positive urgency	x		
**71**	*No name*	Yang & Nan, 2019	RCT	0	x			x						Locomotion & assessment orientation	x		
**72**	Mobile Coach Tobacco & Mobile Coach Tobacco +	Haug et al., 2017a	RCT	6	x				x							x	
**73**	BASICS (Brief Alcohol Screening and Intervention for College	Lee et al., 2010	RCT	6	x		x		x					Familiy history of drug problems		x	

	Students - adaption for marijuana)																		
**74**	CAAT (Cannabis Approach Avoidance Training)	Karoly et al., 2019	RCT	0	x											fMRI: Cannabis cue reactivity	x		
**75**	eCHUG (electronic version of Check-up to go)marijuana	Elliott et al., 2014	RCT	1	x		x			x						Social desirability & AUD symptoms		x	
		Palfai et al., 2014b	RCT	6	x	x	x		x		x					General health behavior		x	
**76**	*No name*	Buckner et al., 2020	RCT	0.5	x											Social anxiety & pos. & neg. affects		x	
**77**	*No name*	Walukevich-Dienst et al., 2019	RCT	1	x		x												x
**78**	iHeLP	Braciszewski et al., 2018	RCT	12	x		x								x	Depressive symptoms		x	
**79**	d-SBI (Digital screening and brief intervention for alcohol misuse)	Sharma et al., 2023	RCT	3	x													x	
**80**	U@Uni:LifeGuide	Cameron et al., 2015	RCT	6	x											Hair sample		x	
**81**	CAAT (Cannabis Approach Avoidance Training)	Jacobus et al., 2018	RCT	0	x			x	x	x			Marijuana		x	Approach bias, withdrawal & craving, stress & reward related symptoms (depression, anxiety)		x	
**82**	MiSARA (substance abuse research assistant)	Coughlin et al., 2021	p-p	1	x	x	x	x								Confidence of change, risk behaviour: past month driving under alc or cannabis, consequences of use, perceived risk, reasons for use, & stress, mood, loneliness, free time, fun, sensation seeking, hopefulness		x	
**83**	myPlaybook	Rulison et al., 2022 (study 1)	Fractional factorial design	1	x	x				x	x	x		x					MOST
		Rulison et al., 2022 (study 2)	Fractional factorial design	1	x	x				x	x	x		x					MOST

		Rulison et al., 2022 (study 3)	Fractional factorial design	0	x	x		x	x	x	x				MOST
**84**	Meine Zeit ohne - Die Challenge	Pietsch et al., 2023	RCT	0	x	x						x	Physical activity, mental health	x	
**85**	Project Fitness	Moore et al., 2012	RCT	0			x	x	x				Prototype images & willingness & behavior coupling	x	

* RCT = randomized designs with control groups, p-p = pre-post designs, qual = qualitative methods

Supplementary file S10_Evidence assessment: Comparison groups

	Project / intervention	Publication	Study design*	No. Study arms	Comparison groups					
					Assess-ment only	Non-digital intervention		Digital interventions		
						Intervention Personally delivered	Other non-digital intervention	A	B	C
**A Adolescents**										
**1**	Alc-Check	Haug et al., 2013	p-p	1						
**2**	Alcohol Alert / Alerta Alcohol	Jander et al., 2016	RCT	2	**X**					
		Martinez-Montilla et al., 2020	RCT	2	**X**					
		Vargas-Martinez et al., 2019	RCT	2	**X**					
**3**	Click City®: Alcohol	Gordon et al., 2017	RCT	2			Standard alc prevention curriculum			
**4**	Climate Schools	Newton et al., 2022a	RCT	4			Preventure (selective for high-risk students) & health education as usual	CAP: climate & Preventure		
		Vogl et al., 2009	RCT	2			Usual classes			
**5**	eCHUG (electronic version of Check-up to go)	Doumas et al., 2019	RCT	2	**X**					
		Doumas et al., 2020a	RCT	2	**X**					
		Doumas et al., 2020b	p-p	1						

		Doumas et al., 2021	RCT	2	**X**				
**6**	HSD (Healthy School and Drugs)	Koning et al., 2011	RCT	4		Regular curriculum	Arm 1: student intervention	Arm 2: combined student-parent intervention	Arm 3: parent intervention
**7**	kiR (keepin' it REAL)	Shin et al., 2018	RCT	2			Rural vs urban version		
**8**	MobileCoach Alcohol	Haug et al., 2017b	RCT	2	**X**				
		Haug et al., 2020	RCT	2	**X**				
		Haug et al., 2023	RCT	1					
**9**	PALMSS (peer alcohol knowledge, low-risk drinking, media-influence, social drinking, self-efficacy)	Hongthon et al., 2016	RCT	2		Alcohol education as usual			
**10**	Smart Choices 4 Teens	Byrnes et al., 2019	RCT	2	**X**				
**11**	SPORT	Mathews et al., 2007	RCT	2			General health information		
**12**	VR *FestLab* (virtual reality FestLab)	Guldager et al., 2022	RCT	2			Other VR game (Oculus Quest - First Steps)		
**13**	WDYD (What do you drink?)	Voogt et al., 2013	RCT	2	**X**				
**14**	*No name*	Ho et al., 2021	RCT	2		Conventional health education via leaflet			
**15**	*No name*	Knight et al., 2018	p-p	1					
**16**	*No name*	Russell et al., 2017	RCT	4			Pos or neg message with or without epilogue (2x2 design)		
**17**	*No name*	Schinke et al., 2009b	RCT	2	**X**				

**18**	C2Q-Teen (Craving to Quit-Teen)	Pbert et al., 2020	RCT	3		Written cessation materials	C2Q-Teen (Craving to Quit-Teen)	QuitSTART smoking appl. (without mindfulness component)
**19**	QuitSTART smoking app	Pbert et al., 2020	RCT	3		Written cessation materials	C2Q-Teen (Craving to Quit-Teen)	QuitSTART smoking appl. (without mindfulness component)
**20**	*No name*	Kong et al., 2017	p-p	1				
**21**	*No name*	Mason et al., 2015	RCT	2			Attention control condition (30 health-based texts)	
**22**	*No name*	Shi et al., 2013	RCT	2		Self-help pamphlet		
**23**	Invite Only VR (virtual reality)	Weser et al., 2021	RCT	2		Education as usual?		
**24**	smokeSCREEN	Bteddini et al., 2023	sequential mixed methods design	3		CATCH My Breath	Educational materials	
**25**	xkpts.com ((reader: “perquèpetes.com”, translation: “why joints?”)	Ariza et al., 2013	quasi-experimental	2	**X**			
**26**	P4T (POP4Teens) vs JTT (JustThinkTwice)	Marsch et al., 2021	RCT	2			active controls: JustThinkTwice website	
**27**	Elm City Story (PlayForward)	Montanaro et al., 2015	RCT	2			Attention-control games	
**28**	REAL media	Greene et al., 2020	RCT	2	**X**			
**29**	*No name*	Fang & Schinke, 2013	RCT	2	**X**			

**30**	E-MOVO (Dutch for Electronic Monitoring and Health Promotion)	Crutzen et al., 2008	p-p	1			
**31**	Health4Life	Champion et al., 2023	RCT	2			Usual health education
**32**	Climate Schools	Champion et al., 2016	RCT	2			Lessons as usual, incl. Drug education
		Newton et al., 2022b	RCT	2			Health education as usual
**33**	*No name*	Gryczynski et al., 2021	RCT	3	**X**	**X**	
**34**	*No name*	Knight et al., 2019	RCT	2			Care as usual
**35**	Healthy School and Drugs	Malmberg et al., 2014	RCT	3		**X**	Regular curriculum
**36**	¿Qu é pasa si te pasas? (What happens if you go to far?)	Fuentes et al., 2023	p-p	1			
**37**	ready4life	Haug et al., 2022	RCT	2	**X**		
**38**	SmartCoach	Paz Castro et al., 2022	RCT	2	**X**		
**39**	RealTeen	Schwinn et al., 2019	RCT	2	**X**		
		Schwinn et al., 2010	RCT	2	**X**		
**40**	Vamos	Schwinn et al., 2021	RCT	2	**X**		
**41**	*No name (family interaction)*	Schinke et al., 2009a	RCT	2	**X**		
		Schinke et al., 2011	RCT	2	**X**		
**42**	*No name*	Schwinn et al., 2015	RCT	2	**X**		
**43**	E-health4Uth	Bannink et al., 2014	RCT	3	**X**		E-health intervention & nurse consultation

**44**	Your Decision Counts TM	Evers et al., 2012	RCT	2	**X**			
**45**	WISEteens	Arnaud et al., 2016	RCT	2	**X**			
**46**	Girls and Stress	Schinke et al., 2005	RCT	2			Conventional group prevention program "Keep a clrear mind"	
**47**	*No name*	Williams et al., 2005	RCT	2	**X**			
**48**	Pure Rush	Stapinski et al., 2018	RCT	2			Standard education	
**49**	Climate Schools Psychostimulant & Cannabis Modlue	Vogl et al., 2014	RCT	2			Drug education as usual	
**50**	Refuse to Use	Duncan et al., 2020	p-p	1				
**51**	Climate Schools: Ecstasy and Emerging Drugs Module	Champion et al., 2018	RCT	2			Lessons as usual, incl. Drug education	
**B Emerging adults**								
**1**	Alcohol 101 Plus™	Carey et al., 2011 (study 2)	RCT	4	**X**		Brief motivational intervention	Alcohol Edu for Sanctions(®)
**2**	Alcohol 101	Barnett et al., 2007	RCT	4		Brief MI with & without booster		Alcohol 101 with & without booster
		Braitman et al., 2016	RCT	2				Alcohol 101 with vs without booster
		Carey et al., 2009	RCT	2			Brief Motivational Interview	
**3**	Alcohol Edu® for Sanctions	Carey et al., 2011 (study 1)	RCT	4	**X**		Brief Motivational Interview	Alcohol 101 Plus™
**4**	Alcohol Edu® for College	Croom et al., 2009	RCT	2	**X**			
		Hustad et al., 2010 (A)	RCT	3	**X**			eCHUG

		Paschall et al., 2014	RCT	2	X				
5	Alcohol-Wise	Croom et al., 2015 (study 1)	RCT	2	X				
		Croom et al., 2015 (study 2)	RCT	2	X				
		Gilbertson et al., 2017	p-p	1					
		Strohman et al., 2016	RCT	2	X				
6	alcooquizz.ch (1)	Bertholet et al., 2015	RCT	2	X				
		Bertholet et al., 2016	RCT	2	X				
		Bertholet et al., 2018	RCT	2	X				
7	BASICS (Brief Alcohol Screening and Intervention for College Students)	Lee et al., 2014	RCT	5	X	X	Intervention in-person with friend	WebBASICS with friend	
		Linowski et al., 2016	RCT	2	X				
		Murphy et al., 2010, (study 1)	RCT	3				ALCOHOL 101 Plus	
		Murphy et al., 2010 (study 2)	RCT	2	X			eCHUG	
		Patrick et al., 2014	RCT	2	X				
		Patrick et al., 2021	RCT	5	X			Sequential multiple assignment	
		Saitz et al., 2007	RCT	3	X			Minimal vs more extensive intervention	
8	BASICS (Brief Alcohol Screening and Intervention for College Students) - PNF component	LaBrie et al., 2013	RCT	10	X			WebBASICS	8 specific reference groups
		Larimer et al., 2021	RCT	2	X				
		Moreira et al., 2012	RCT	3	X			Delayed control group	

		Neighbors et al., 2004	RCT	2	X					
		Neighbors et al., 2006	RCT	2	X					
		Neighbors et al., 2010	RCT	5			Attention control: non-drug-related facts about students at university	PNF (BASICS) biannual non-gender specific	PNF (BASICS) single gender-specific	PNF (BASICS) single non-gender specific
**9**	Campus GANDR (Gamified Alcohol Norm Discovery & Readjustment)	Boyle et al., 2017	RCT	2				Standard PNF		
**10**	CDCU (College Drinker's Check-up)	Carey et al., 2017	RCT	2	X					
		Hester et al., 2012 (study 1)	RCT	2	X					
		Hester et al., 2012 (study 2)	RCT	2	X					
**11**	CIAN (Call it a Night®)	Fucito et al., 2017	RCT	2			Healthy Behaviors'			
**12**	College Alc	Bersamin et al., 2007	RCT	4	X			Drinkers vs non-drinkers		
**13**	D_ARIANNA ((Digital-Alcohol Risk Alertness Notifying Network for Adolescents and Young Adults))	Carrà et al., 2016	p-p	1	X					
**14**	DrAFT-CS (Drinking Assessment and Feedback Tool for College Students)	Wagener et al., 2012	RCT	4	comprehensive & minimal	X				
		Weaver et al., 2014	RCT	4	X			Intervention & moderation skills (DrAFT-CS+	Moderation skills	

**15**	ECALC (expectancy challenge alcohol literacy curriculum)	Dunn et al., 2020 (B) 2019?	Outcome comparison study; random assignment	2		BMI & PNF (BASICS session)		
**16**	eCHUG (electronic version of Check-up to go)	Alfonso et al., 2012	RCT	3		BASICS (individual)) & CHOICES (group)		
		Braitman et al., 2022	RCT	3			Additional booster with normative feedback	Additional booster with normative feedback & personal behavioural strategies
		Doumas et al., 2011	RCT	2			Education	
		Hustad et al., 2010 (B)	RCT	3	**X**		Alcohol EDU	
		Thompson et al., 2018	RCT	2	**X**			
		Tahaney & Palfay, 2017	RCT	3	**X**		Web-based intervention without text messages	
		Walters et al., 2007	RCT	2	**X**			
**17**	eentjeteveel	Fraeyman et al., 2012	qual	1				
**18**	eSBI (electronic screening and brief intervention)	Kypri et al., 2013	RCT	2	**X**			
		Kypri et al., 2014	RCT	2	**X**			
		Kypri et al., 2008	RCT	3		information pamphlet	Multi-dose intervention	

**19**	ETUCARE	Theurel et al., 2022	Non-radomized controlled trial	2	**X**					
**20**	FIT (functional imagery training)	Shuai et al., 2022	RCT	2				Standard risk information		
**21**	M-PASS (Michigan Prevention and Alcohol Safety for Students)	Bingham et al., 2011	Paired intervention-control design	2	**X**					
**22**	MyStudentBody:Alcohol	Chiauzzi et al., 2005	RCT	2				Acohol and You (educational content)		
**23**	myPlaybook	Fearnow-Kenney et al., 2016	RCT	2	**X**					
		Zamboanga et al., 2019	RCT	2		**X**				
**24**	PantherTRAC	Suffoletto et al., 2016	p-p	1						
**25**	PartyWise	Schwarz et al., 2022	RCT	2			Attention control			
**26**	Ray's Night Out	Hides et al., 2018	RCT	2		**X**				
		Pocuca et al., 2016	Mixed methods	1						
**27**	THRIVE (Tertiary Health Research Intervention via Email)	Kypri et al., 2009	RCT	2	**X**					
		Leeman et al., 2016	RCT	4	**X**			THRIVE with indirect PBS only	THRIVE with direct PBS only	THRIVE with direct and indirect PBS
**60**	Unitcheck	Bewick et al., 2013	RCT	2	**X**					
		Marley et al., 2016	qual	1						
**29**	WDYD (What do you drink?)	Voogt et al., 2014a	RCT	2	**X**					

**30**	Y1-CAP (Year 1 College Alcohol Profile)	Shell et al., 2019	Completers vs non-completers	2			Non-completers		
**31**	No name	Bernstein et al., 2018	RCT	2	**X**				
**32**	No name	Bonar et al., 2022	RCT	3			Intervention plus monetary incentives	Attention-placebo control	
**33**	*No name*	Braitman et al., 2020	RCT	3			Alcohol 101 plus	General health education (Better Health)	
**34**	*No name*	Boyle et al., 2021	RCT	3			Traditional web-based PNF	Gamified only PNF	Social media-inspired gamified
**35**	*No name*	Bryant et al., 2013	RCT	2			Generic feedback		
**36**	*No name*	Buckner et al., 2019	RCT	2			Non-drinking control-feedback		
**37**	*No name*	Butler et al., 2009	RCT	3		**X**			
**38**	*No name*	Carey et al., 2020	RCT	2			Messages about historical facts		
**39**	*No name*	Collins et al., 2014	RCT	3	**X**		PNF		
**40**	*No name*	Doumas et al., 2009	RCT	2		**X**			
**41**	*No name*	Ellis et al., 2017	RCT	2			Absence vs presence of empathic narrator		
**42**	*No name*	Gex et al., 2023	RCT	2			Education		
**43**	*No name*	Glowacki et al., 2020	RCT	3			General health information	Messages matching regulatory focus	Messages mismatching regulatory focus

**44**	*No name*	Hagger et al., 2012	RCT	4	**X**		Implementation intention condition	Mental simulation condition	Implementation intention & mental simulation condition
**45**	*No name*	Hendershot et al., 2010	RCT	2			Attention control feedback		
**46**	*No name*	Kazemi et al., 2020 (study 1)	RCT	2		In-person BMI			
**47**	*No name*	Kazemi et al., 2020 (study 2)	RCT	2	**X**				
**48**	*No name*	LaBrie et al., 2019	RCT	4			Arms 1: gamified PNF only	Arms 2-3: gamified PNF + Visual Copresence & Gamified PNF + Maximum Copresence	Arm 4: standard PNF
**49**	*No name*	Larimer et al., 2023	RCT	5			Arm 1: non-alcohol-related feedback	Arms 2-4: descriptive PNF, injunctive PNF, descriptive & injunctive PNF	Arm 5: mulitcomponent personalized feedback
**50**	*No name*	Leary et al., 2022	RCT	3	**X**		Arm 1: postive messages about people who do not consume	Arm 2: negative messages about people who consume	
**51**	*No name*	Leavens et al., 2020	RCT	3	**X**		Arm 1: typical feedback on PBS	Arm 2: on descriptive normative feedback on PBS	
**52**	*No name*	Lewis et al., 2007	RCT	3	**X**		Gender-specific feedback	Gender-neutral feedback	
**53**	*No name*	Ma, 2022	RCT	2			Delivery via tablet	Delivery via hmd	

**54**	*No name*	Mason et al., 2014	RCT	2	**X**		
**55**	No name	McGeary et al., 2014	RCT	2		Attention control	
**56**	*No name*	Neighbors et al., 2009	RCT	2	**X**		
**57**	*No name*	Norman et al., 2018	RCT	8		2 (self-affirmation) x 2 (TPB messages) x 2 (implementation intention)	
**58**	*No name*	Palfai et al., 2011	RCT	4 (2x2 design)		Alcohol feedback vs. no alcohol feedback	Motivational Assessment vs. No Motivational Assessment
**59**	*No name*	Palfai et al., 2014a	RCT			Feedback about other health-related behaviour	
**60**	*No name*	Pilling et al., 2007	RCT	4		3 different types of persuasive messages	Neutral' message to discourage heavy drinking)
**61**	*No name*	Ridout & Cambell, 2014	RCT	2	**X**		
**62**	*No name*	Riordan et al., 2023	RCT	3	**X**	web-based interventions	Web-based interventions + ecological momentary interventions
**63**	*No name*	Savage et al., 2015	RCT	3	**X**	SOTA (state of the art) intervention	
**64**	*No name*	Schuckit et al., 2016	RCT	3	**X**	SOTA (state-of-the-art) condition: information withour emphasis on level of risk	

**65**	*No name*	Smallman et al., 2023	RCT	3		Sham (negative event with detailed description)	Negative event only	
**66**	*No name*	Spijkerman et al., 2010	RCT	3	**X**	Normative feedback additionally		
**67**	*No name*	Tello et al., 2018	RCT	2		Neutral images for alcohol		
**68**	*No name*	Thombs et al., 2007	RCT	2		BAC readings only		
**69**	*No name*	Walters et al., 2009	RCT	4	**X**	MI	MI with feedback	Feedback only
**70**	*No name*	Whitt et al., 2019	RCT	2	**X**			
**71**	*No name*	Yang & Nan, 2019	RCT	2		Arm 1: descriptive norms	Arm 2: Injunctive norms	
**72**	Mobile Coach Tobacco & Mobile Coach Tobacco +	Haug et al., 2017a	RCT	2	**X**			
**73**	BASICS (Brief Alcohol Screening and Intervention for College Students - adaption for marijuana)	Lee et al., 2010	RCT	2	**X**			
**74**	CAAT (Cannabis Approach Avoidance Training)	Karoly et al., 2019	RCT	2		CAAT-sham training		
**75**	eCHUG (electronic version of Check-up to go)marijuana	Elliott et al., 2014	RCT	4	brief & extended	Brief intervention	Extended intervention	
		Palfai et al., 2014b	RCT	4		Feedback about other health-related behaviour	Onsite vs off-site	
**76**	*No name*	Buckner et al., 2020	RCT	2	**X**			
**77**	*No name*	Walukevich-Dienst et al., 2019	RCT	2		PNF (personal normative feedback only)		

**78**	iHeLP	Braciszewski et al., 2018	RCT	2		Contact control
**79**	d-SBI (Digital screening and brief intervention for alcohol misuse)	Sharma et al., 2023	RCT	2		Brief education
**80**	U@Uni:LifeGuide	Cameron et al., 2015	RCT	2	**x**	
**81**	CAAT (Cannabis Approach Avoidance Training)	Jacobus et al., 2018	RCT	2		CAAT-sham training ; same as intervention, but without contingency
**82**	MiSARA (substance abuse research assistant)	Coughlin et al., 2021	p-p	1		
**83**	myPlaybook	Rulison et al., 2022 (study 1)	Fractional factorial design	16 conditions		6 different modules
		Rulison et al., 2022 (study 2)	Fractional factorial design	8 conditions		4 different modules
		Rulison et al., 2022 (study 3)	Fractional factorial design	8 conditions		5 different modules
**84**	Meine Zeit ohne - Die Challenge	Pietsch et al., 2023	RCT	2	**x**	
**85**	Project Fitness	Moore et al., 2012	RCT	2		Standard care

* RCT = randomized designs with control groups, p-p = pre-post designs, qual = qualitative methods
